# Importin α2 participates in RNA interference against bamboo mosaic virus accumulation in *Nicotiana benthamiana* via NbAGO10a‐mediated small RNA clearance

**DOI:** 10.1111/mpp.13422

**Published:** 2024-01-19

**Authors:** Jiun‐Da Wang, Yau‐Heiu Hsu, Yun‐Shien Lee, Na‐Sheng Lin

**Affiliations:** ^1^ Institute of Plant and Microbial Biology Academia Sinica Taipei Taiwan; ^2^ Graduate Institute of Biotechnology National Chung Hsing University Taichung Taiwan; ^3^ Department of Biotechnology Ming Chuan University Taipei Taiwan

**Keywords:** AGO10, BaMV, importin α, RNA silencing, vsiRNAs

## Abstract

Karyopherins, the nucleocytoplasmic transporters, participate in multiple RNA silencing stages by transporting associated proteins into the nucleus. Importin α is a member of karyopherins and has been reported to facilitate virus infection via nuclear import of viral proteins. Unlike other RNA viruses, silencing of importin α2 (α2*i*) by virus‐induced gene silencing (VIGS) boosted the titre of bamboo mosaic virus (BaMV) in protoplasts, and inoculated and systemic leaves of *Nicotiana benthamiana*. The enhanced BaMV accumulation in importin α2*i* plants was linked to reduced levels of RDR6‐dependent secondary virus‐derived small‐interfering RNAs (vsiRNAs). Small RNA‐seq revealed importin α2 silencing did not affect the abundance of siRNAs derived from host mRNAs but significantly reduced the 21 and 22 nucleotide vsiRNAs in BaMV‐infected plants. Deletion of BaMV TGBp1, an RNA silencing suppressor, compromised importin α2*i*‐mediated BaMV enhancement. Moreover, silencing of importin α2 upregulated NbAGO10a, a proviral protein recruited by TGBp1 for BaMV vsiRNAs clearance, but hindered the nuclear import of NbAGO10a. Taken together, these results indicate that importin α2 acts as a negative regulator of BaMV invasion by controlling the expression and nucleocytoplasmic shuttling of NbAGO10a, which removes vsiRNAs via the TGBp1‐NbAGO10a‐SDN1 pathway. Our findings reveal the hidden antiviral mechanism of importin α2 in countering BaMV infection in *N*. *benthamiana*.

## Graphical abstract

Silencing of importin α's enhances BaMV accumulation but reduces its virus‐derived small‐interfering RNAs (vsiRNAs) via NbAGO10a and SDN1‐mediated small RNA clearance in *Nicotiana benthamiana*.

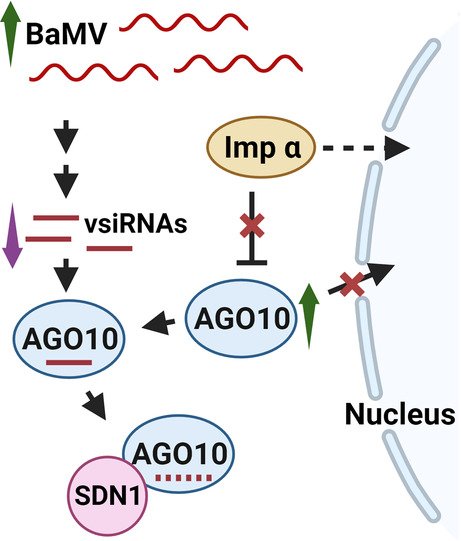

## 1 INTRODUCTION

Karyopherins are a protein family that regulates protein trafficking through the nuclear pore that is surrounded by the nuclear pore complex (NPC). Nuclear transport is determined by the signal peptides hosted by cargo proteins, including the nuclear localization signal (NLS) that is bound by importins for nuclear import, as well as the nuclear export signal recognized by exportins for cargo protein export (Chook & Blobel, 2001). In the classical nuclear import pathway, NLS‐hosting proteins are bound by importin α to form protein complexes with importin β. Then, importin β interacts with the FG repeat region of nucleoporins in the NPC to facilitate nuclear import of the cargo‐importin α/β complex. In the nucleus, binding of Ran‐GTP with importin β triggers dissociation of the complexes to release the cargos. Finally, the importins are exported into the cytosol by exportins for another round of nuclear transport (Chook & Blobel, 2001; Chook & Suel, 2011; Goldfarb et al., 2004).

RNA silencing is a primary defence mechanism against RNA virus infection in plants. Most RNA viruses replicate in the host cytoplasm (Carbonell et al., 2016), with the newly synthesized double‐stranded RNAs (dsRNAs) of viruses being processed by host‐derived Dicer‐like proteins (DCLs), such as DCL2 and DCL4, to generate virus‐derived small‐interfering RNAs (vsiRNAs). These vsiRNAs are then loaded into ARGONAUTE (AGO) proteins to form an RNA‐induced silencing complex (RISC) that targets the viral genomic RNAs (gRNAs) for RNA silencing by translational inhibition or RNA cleavage (Leonetti et al., 2021; Zhang et al., 2015). Moreover, with the assistance of Suppressor of gene silencing 3 (SGS3), the processed RNAs can be catalysed by RNA‐dependent RNA polymerases (RDRs), such as RDR6, to synthesize dsRNAs, which are subsequently cleaved by DCLs to generate secondary siRNAs and amplify RNA silencing cascades (Devert et al., 2015; Kumakura et al., 2009; Lopez‐Gomollon & Baulcombe, 2022; Vazquez & Hohn, 2013).

The nuclear transport of viral proteins mediated by the importin α family plays a central role in viral infection (Fulcher & Jans, 2011). For example, the viral suppressor of RNA silencing (VSR) of cauliflower mosaic virus (CaMV), P6, is imported into the nucleus by importin α where it associates with DRB4 to suppress the RNA silencing pathway in *Nicotiana benthamiana* (Haas et al., 2008). Beet black scorch virus (BBSV) movement protein P7a accumulates in nucleoli and Cajal bodies via an importin α‐dependent transport system. Mutation of the R‐rich motif of P7a was found to limit its interaction with the nucleolar protein, fibrillarin, and it reduced levels of BBSV accumulation in *N. benthamiana* protoplasts (Wang et al., 2012). Moreover, downregulation of *N. benthamiana* importin α1 and α2 by means of virus‐induced gene silencing (VIGS) reduced systemic movement of potato mop‐top virus (PMTV) (Lukhovitskaya et al., 2015). Several studies have indicated that importin β isoforms not only transport nuclear cargos, but also participate in various RNA silencing pathways in planta. For instance, *Arabidopsis* KETCH1 regulates HYL1 transport from the cytoplasm to the nucleus for miRNA biogenesis (Zhang et al., 2017). Two *Arabidopsis* importin β isoforms, SAD2/EMA1 and TRANSPORTIN1 (TRN1), respectively negatively and positively regulate the loading of small RNAs into AGO1 complexes (Cui et al., 2016; Wang et al., 2011).

Bamboo mosaic virus (BaMV) is a well‐studied member of the genus *Potexvirus*. Its virion displays a flexuous filamentous morphology and harbours a single‐stranded, positive‐sense RNA genome (DiMaio et al., 2015; Lin et al., 1994). The BaMV genome is approximately 6.4 kb in length and encodes five open reading frames (ORF1–5) (Lin et al., 1994). ORF1 encodes a 155 kDa replicase with three functional domains, including the N‐terminal capping enzyme domain, the helicase‐like domain, and the RNA‐dependent RNA polymerase domain in the C‐terminal region (Huang et al., 2004; Li et al., 1998, 2001). ORFs 2 to 4 encode movement proteins, namely the triple gene block (TGB) p1 to p3. TGBp1 localizes in the host nucleus and cytoplasm and displays VSR activity and RNA‐binding ability for BaMV movement (Chang et al., 1997; Huang et al., 2019; Lin et al., 2004). TGBp2 is an endoplasmic reticulum (ER)‐associated transmembrane protein and exhibits nonspecific RNA‐binding ability (Liou et al., 2015). Like TGBp2, TGBp3 is an ER‐associated membrane protein and it co‐operates with TGBp2 to guide BaMV ribonucleoprotein (RNP) complexes to plasmodesmata for cell‐to‐cell movement (Liou et al., 2015). ORF5 encodes coat protein (CP) that accumulates in the host nucleus and cytoplasm, and it is responsible for BaMV encapsidation, cell‐to‐cell movement and development of viral infection symptoms (Hung et al., 2014; Lan et al., 2010).

In this study, we demonstrate that importin α isoforms negatively regulate BaMV infection in *N*. *benthamiana*, unlike for other RNA viruses. Silencing of importin α1 or α2 enhances BaMV infection and reduces the accumulation of RDR6‐dependent secondary vsiRNAs. By means of RNA‐sequencing (RNA‐seq) analysis of *N*. *benthamiana* leaves, we show that NbAGO10a is induced by importin α2 silencing following BaMV infection and that nuclear accumulations of 3HA‐mCherry‐NbAGO10a are reduced in importin α2‐silenced plants. Because NbAGO10a is involved in BaMV vsiRNA sequestration and degradation, we postulate that importin α2 regulates NbAGO10a homeostasis and localization to modulate host RNA silencing defence against BaMV infection.

## 2 RESULTS

### 2.1 Enhanced BaMV infection in importin α1‐ or α2‐silenced *N*. *benthamiana*

The 14 importin α isoforms of *N*. *benthamiana* have been grouped previously into three clades: clade I comprises 10 isoforms, clade II has two, and clade III a further two (Figure S1a) (Lukhovitskaya et al., 2015; Perez‐Canamas & Hernandez, 2018). To explore the functions of these importin α isoforms in BaMV infection, we used VIGS technology to knock down expression of importin clade I (α1 and α2), clade II (αc2) or clade III (αc3) isoforms in *N*. *benthamiana*. Individual expression levels of importin α1, α2, αc2 and αc3 were strongly reduced upon agroinfiltration of *N*. *benthamiana* leaves with tobacco rattle virus (TRV)‐based vectors (Senthil‐Kumar & Mysore, 2014) (Figure S1c–f), although VIGS of importin α2 also slightly reduced the expression of importin α1 (Figure S1c–f). The phenotypes of importin α‐silenced (αs*i*) plants did not differ from control mCherry*i* plants, except that we observed a dwarf phenotype in the importin α2*i* plants (Figure S1b). Similar dwarf phenotypes have been reported previously for other importin α‐related mutants, such as *Arabidopsis mos6* (importin α3), *gir1* (importin β‐like protein) and *importin α1*/*α2* double mutants (Ludke et al., 2021; Panda et al., 2020).

**FIGURE 1:**
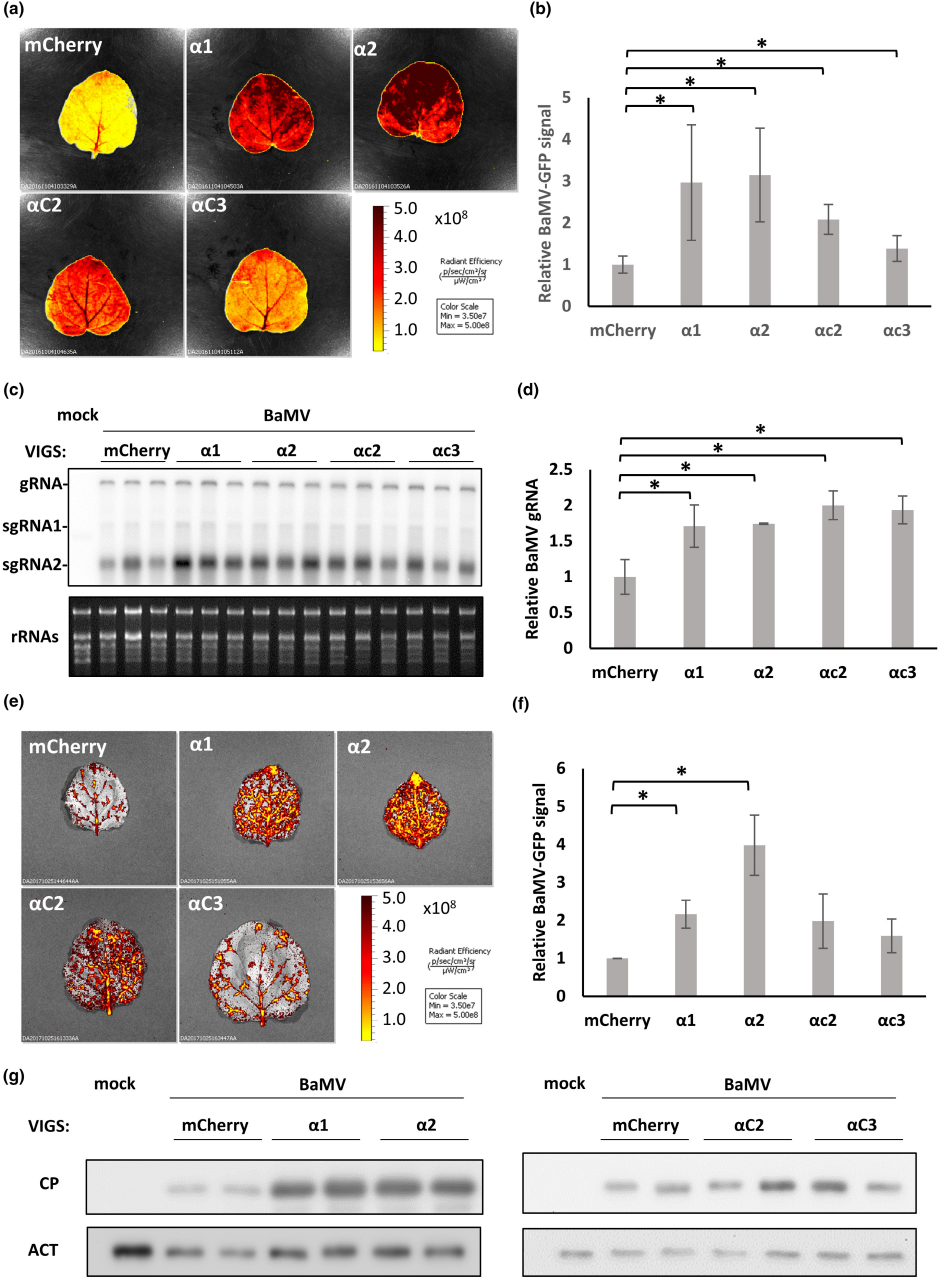
Accumulation of BaMV in importin α isoform‐silenced *Nicotiana benthamiana*. Virus‐induced gene silencing (VIGS) of importin α isoforms was carried out by agroinfiltration of 18‐day‐old plants with indicated plasmids. At 7 days after VIGS, the upper leaves of silenced plants were infected by agroinfiltration with the BaMV infectious plasmids pKBG (a, b, e, f) or pKB (c, d, g). (a, b, e, f) GFP fluorescence signal of BaMV‐GFP in inoculated leaves (ILs) at 5 days post‐infiltration (dpi) (a, b) or systemic leaves (SLs) at 14 dpi (e, f), respectively, which were harvested, measured and quantified using an IVIS imaging system. (b, f) Statistical analysis of GFP signals from three independent biological samples of ILs (b) and SLs (f). Values have been normalized against those in mCherry‐silenced plants. Data represent mean ± *SD* and were analysed by Student's *t* test. *p < 0.05. Similar results were obtained from three independent experiments. Pseudo‐colour scale bars of eGFP intensity are presented in (a) and (e). (c, d) Accumulations of BaMV in the ILs of silenced plants, as detected by RNA blotting at 5 dpi (c), with BaMV gRNA quantified from three independent samples using ImageJ (d). Similar results were obtained from three independent experiments. Ribosomal RNA was used as an internal control. gRNA, genomic RNA; sgRNA, subgenomic RNA. (g) Accumulation of BaMV coat protein (*CP*), as determined by immunoblotting with anti‐CP antibody at 14 dpi. *Actin* (ACT) was used as an internal control.

To visualize the effects of importin α silencing on BaMV accumulation, we introduced the plasmids harbouring GFP‐tagged BaMV (BaMV‐GFP) (Prasanth et al., 2011) into the importin α‐silenced leaves of *N. benthamiana* through agroinfiltration. Following agroinfiltration of BaMV‐GFP, we easily determined a 3‐fold increase in green fluorescent protein (GFP) signal intensity for BaMV‐GFP in inoculated leaves (ILs) of α1*i* and α2*i* plants, a 2‐fold increase for αc2*i* plants, and a 1.4‐fold increase for αc3*i* plants, compared to control mCherry*i* plants at 5 days post‐infiltration (dpi) (Figure 1a,b). Through agroinfiltration with wild‐type (WT) BaMV, RNA blotting revealed accumulation (2‐fold relative to control plants) of viral genomic RNA (gRNA) in individual importin αs*i* plants at 5 dpi (Figure 1c,d). These results indicate that importin α's negatively participate in BaMV and BaMV‐GFP accumulation. Moreover, we found that BaMV‐GFP signal and CP accumulation were enhanced in the upper leaves of importin α1*i* or α2*i* plants, but not in those of αc2*i* or αc3*i* plants, at 14 dpi (Figure 1e–g). Taken together, these results reveal that importin αs negatively regulate BaMV accumulation in the ILs of *N*. *benthamiana*, yet silencing of importin α1 or α2 alone enhances BaMV accumulation in systemic leaves (SLs).

### 2.2 Silencing of importin α1 or α2 enhances BaMV accumulation in protoplasts

**FIGURE 2:**
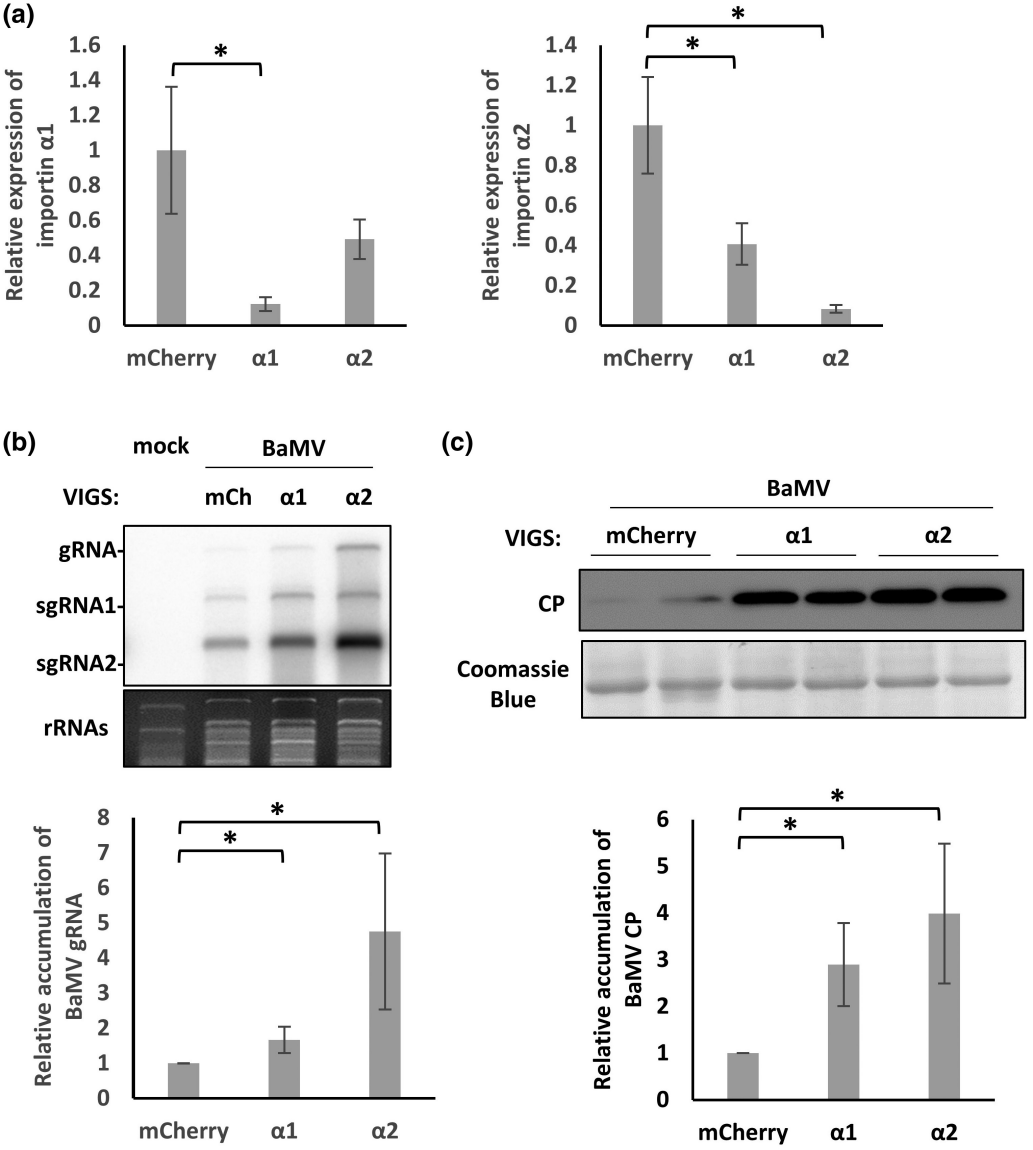
Accumulations of importin αs and BaMV in importin α1‐ or α2‐silenced protoplasts. Protoplasts were isolated from mCherry‐, importin α1‐ or α2‐silenced *Nicotiana benthamiana* leaves at 9 days after virus‐induced gene silencing. The protoplasts were transfected with or without the infectious pCB plasmid of BaMV, and then harvested 20 h post‐inoculation. (a) Expression levels of importin α1 and α2, as determined by reverse transcription‐quantitative PCR (*p < 0.05, *n* = 3, Student's *t* test). (b) RNA blots and statistical analysis of viral RNAs of BaMV. BaMV gRNAs were quantified by ImageJ and normalized to rRNAs (*p < 0.05, *n* = 3, Student's *t* test). (c) Immunoblotting and statistical analysis of BaMV coat protein (CP) (*p < 0.05, *n* = 3, Student's *t* test). BaMV CP was quantified by ImageJ and normalized to total proteins stained with Coomassie blue. gRNA, genomic RNA; sgRNA, subgenomic RNA.

To distinguish if the BaMV accumulation elicited by silencing of importin α1 or α2 is due to virus replication or movement, we assessed the level of BaMV accumulation in the protoplasts of importin α1*i* or α2*i N*. *benthamiana* at 20 h post‐transfection of BaMV pCB plasmid (Lin et al., 2004). The expression of importin α1 or α2 was significantly reduced by about 90% in the isolated protoplasts of α1‐ or α2‐silenced *N*. *benthamiana*, respectively (Figure 2a), although silencing of importin α1 also reduced the expression of importin α2 (Figure 2a). Because of the high identity of importin α1 and α2 (about 80% protein identity) (Lukhovitskaya et al., 2015; Perez‐Canamas & Hernandez, 2018), it may result in potential off‐target effects of VIGS when silencing these importin αs. Moreover, it is worth noting that the silencing efficacy and off‐target effects might vary across different cellular systems, such as silenced leaves (Figure S1c,d) or protoplasts (Figure 2a).

RNA blotting revealed that levels of BaMV gRNA were enhanced about 1.7‐fold or about 5.8‐fold in the α1*i* or α2*i* protoplasts, respectively, relative to mCherry*i* protoplasts (Figure 2b). Immunoblotting also confirmed that levels of BaMV CP increased about 7‐fold or about 10‐fold in the α1*i* or α2*i* protoplasts compared with mCherry*i* protoplasts (Figure 2c). These results indicate that importin α1 and α2 directly affect viral replication at the single cell level.

### 2.3 BaMV vsiRNA accumulations are reduced upon silencing importin α1 or α2

**FIGURE 3:**
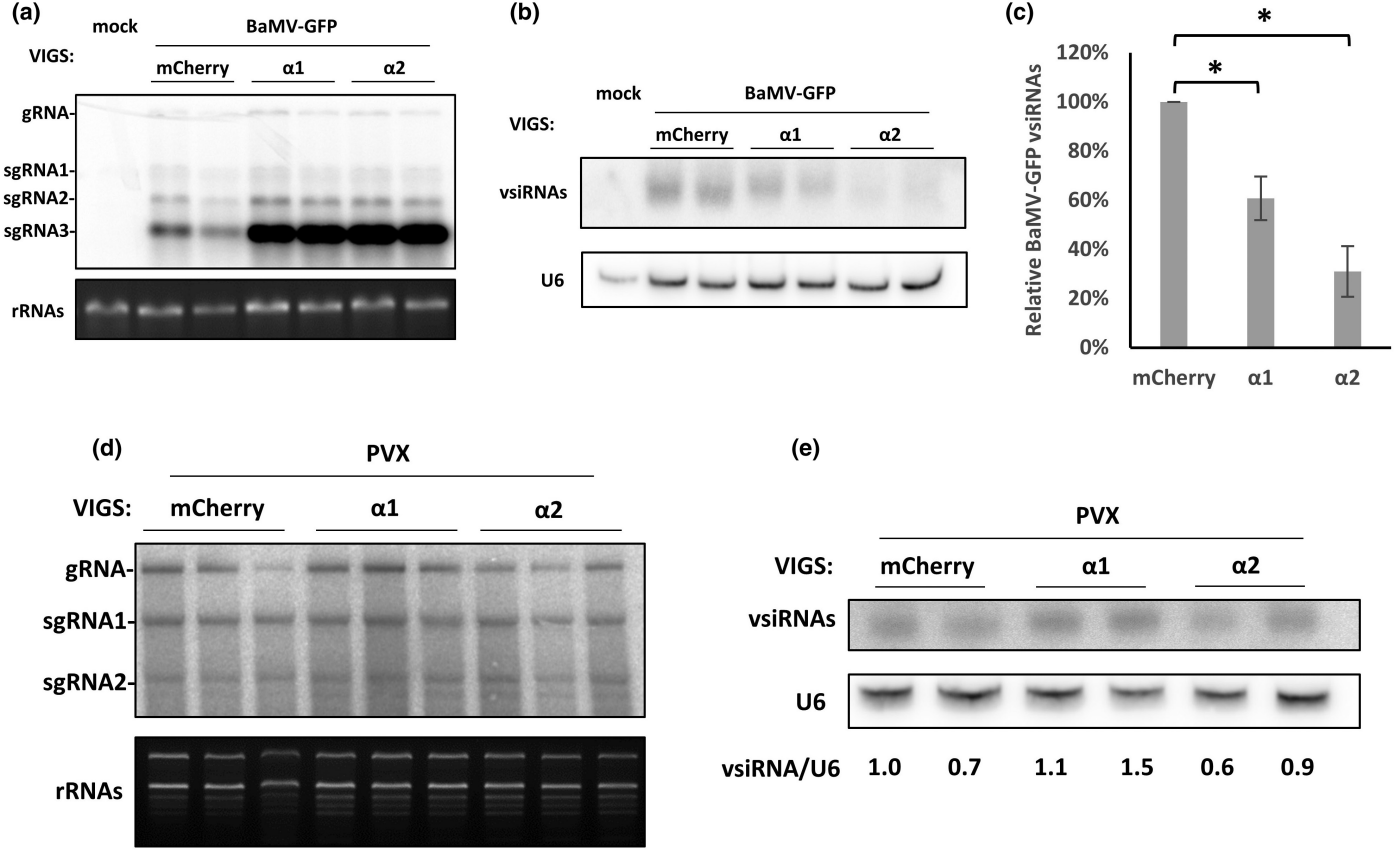
Accumulations of BaMV‐GFP or PVX viral RNAs and vsiRNAs in importin α1‐ or α2‐silenced plants. Virus‐induced gene silencing and viral infection were performed as described in Figure 1. Agroinfiltration with pKBG (a–c) or pKnPVX (d, e) was used separately. (a) RNA blot of BaMV‐GFP viral RNAs. (b) RNA blot of BaMV‐GFP vsiRNAs. (c) Statistical analysis of BaMV vsiRNA accumulation from three independent tests (*p < 0.05, *n* = 3, Student's *t* test). (d) RNA blot of PVX viral RNAs. (e) RNA blot of PVX vsiRNAs. Blots were quantified using ImageJ. U6 was used as an internal control. gRNA, genomic RNA; sgRNA, subgenomic RNA; vsiRNAs, virus‐derived small‐interfering RNAs.

RNA silencing is the major antiviral defence mechanism in plants. We wondered if importin α1 and α2 participate in RNA silencing to tackle BaMV infection. As expected, we found that levels of BaMV‐GFP viral RNAs were enhanced considerably upon silencing of importin α1 or α2 (Figure 3a), but RNA blotting demonstrated that BaMV‐GFP vsiRNAs declined by about 40% or 60% in importin α1*i* or α2*i* plants relative to mCherry*i* plants (Figure 3b,c). To assess whether importin α1 or α2 silencing has similar effects on replication of another potexvirus, we performed similar experiments on potato virus X (PVX). Unlike BaMV, accumulations of PVX and its vsiRNAs were not affected in importin α1*i* or α2*i* plants (Figure 3d,e). These results suggest that the increased levels of BaMV, rather than PVX, triggered by the silencing of importin α1 or α2 could be associated with the decreased levels of BaMV vsiRNAs.

**FIGURE 4:**
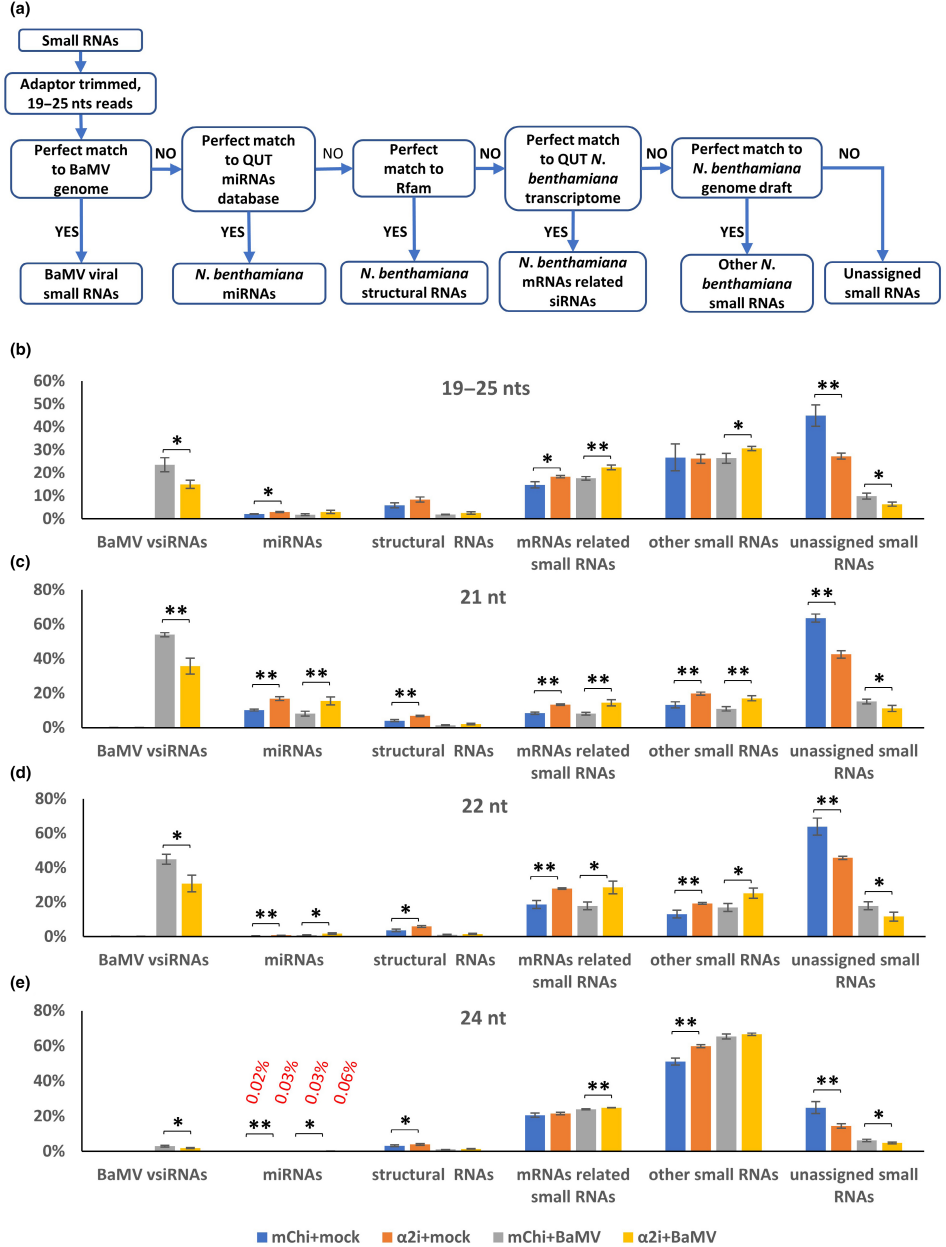
Small RNA profiles of BaMV‐infected and/or importin α2‐silenced *Nicotiana benthamiana*. Virus‐induced gene silencing and BaMV infection with pKB by agroinfiltration were performed as described in Figure 1. (a) Pipeline of the small RNA‐seq analysis. The trimmed reads of 19–25 nucleotides (nt) were used as a query to search against the BaMV genome, the QUT miRNAs database, Rfam, the *N*. *benthamiana* transcriptome, and the QUT *N*. *benthamiana* genome draft. (b–e) Relative accumulations of 19–25 nt (b), 21 nt (c), 22 nt (d), and 24 nt (e) small RNAs (*p < 0.05, **p < 0.01, *n* = 3, Student's *t* test).

To provide a more global view of how small RNAs are regulated by importin α2, we extracted small RNAs from mCherry*i* or importin α2*i N*. *benthamiana* infected with or without BaMV at 5 dpi for small RNA‐seq analysis. Adopting a pipeline to identify various small RNAs (as depicted in Figure 4a), we retrieved approximately 140 million raw reads from each sample (Table S1). Overall, about 20% of BaMV vsiRNA reads in the infected *N*. *benthamiana* samples perfectly matched with the BaMV genome (Table S1). Additionally, 2%–4% of miRNAs and 15%–22% of mRNA‐related siRNAs were identified from *N*. *benthamiana* databases. However, approximately 30% of small RNAs remained unassigned due to the incompleteness of the databases (Figure 4). Upon BaMV infection, 21‐ and 22‐nucleotide (nt) vsiRNAs were predominant, together constituting nearly all of the detected vsiRNAs, although trace amounts of 24‐nt vsiRNAs were also detected in these samples (Figure 4b–e). Notably, we observed a significant reduction (from 24% to 15%) in the overall abundance of BaMV vsiRNAs in BaMV‐infected importin α2*i* plants. This pattern of decline was consistent for the 21‐, 22‐ and 24‐nt vsiRNAs. Our small RNA blotting data confirmed these results (Figure 3b,c). Although levels of BaMV vsiRNAs in the importin α2*i* plants were reduced, importin α2 silencing did not prompt any changes in vsiRNA hotspots when they were mapped onto both the positive‐ and negative‐strand viral RNAs (Figure S2). Moreover, in contrast to vsiRNAs, silencing of importin α2 enhanced the accumulations of 19–25‐nt mRNA‐related siRNAs, miRNAs and other small RNAs, regardless of BaMV infection (Figure 4b–e). Taken together, these results imply differential regulation controlling the generation of vsiRNAs and siRNAs in *N*. *benthamiana* after importin α2 silencing.

### 2.4 BaMV secondary vsiRNAs are reduced in importin α2*i N*. *benthamiana*

**FIGURE 5:**
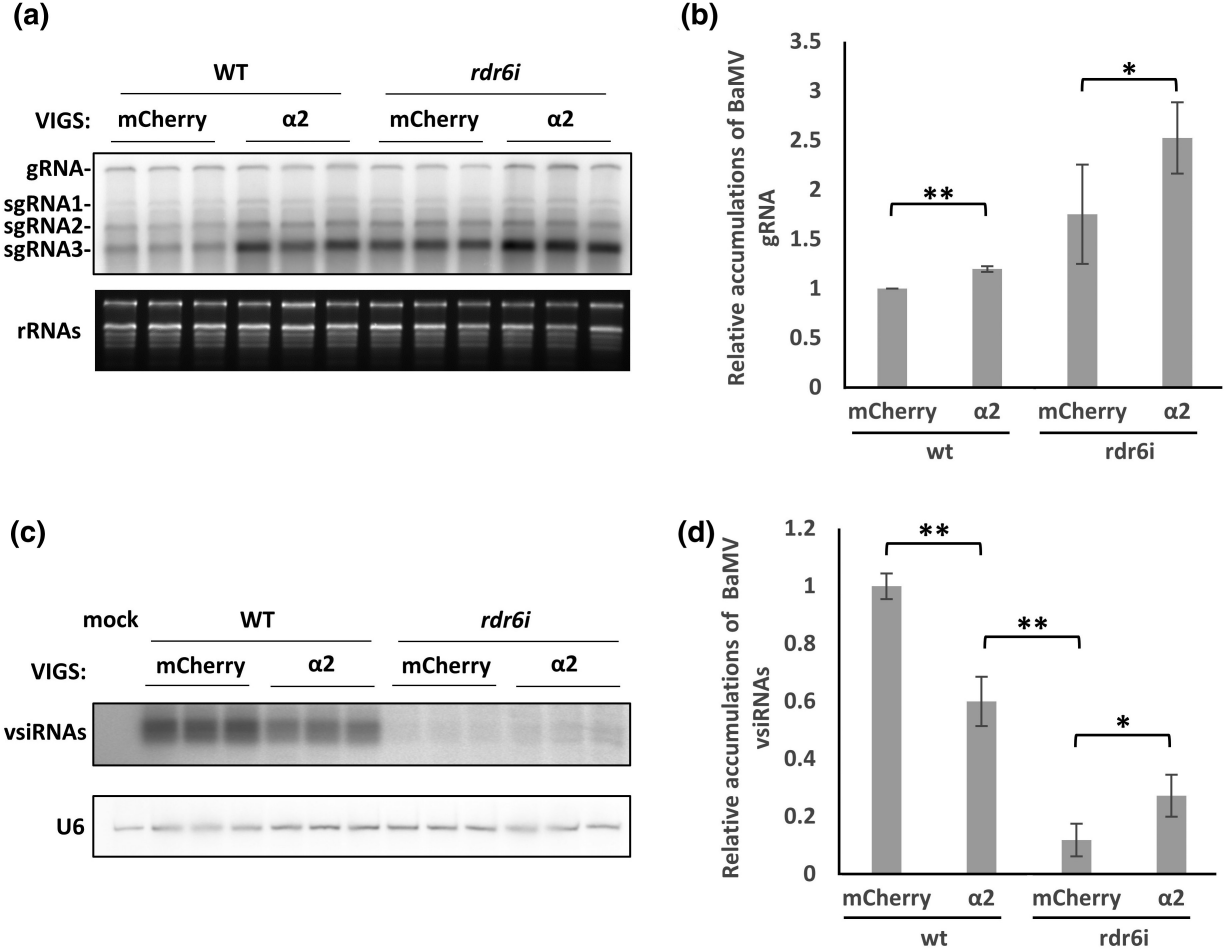
The effects of RDR6 in importin α2‐regulated BaMV‐GFP vsiRNA accumulations. Virus‐induced gene silencing and viral infection (pKBG) of wild‐type (WT) and NbRDR6*i* transgenic *Nicotiana benthamiana* were performed as described in Figure 1. (a) RNA blot of BaMV‐GFP viral RNAs at 5 days post‐inoculation. rRNA was used as the internal control. (b) Statistical analysis of BaMV gRNA from three independent tests (*p < 0.05, *n* = 3, Student's *t* test). (c) RNA blots of BaMV‐GFP vsiRNAs. (d) Statistical analysis of BaMV vsiRNA accumulations (*p < 0.05, *n* = 3, Student's *t* test). Similar results were obtained from three independent tests. Blots were quantified using ImageJ. U6 was used as an internal control. gRNA, genomic RNA; sgRNA, subgenomic RNA; vsiRNAs, virus‐derived small‐interfering RNAs.

Amplification of the secondary siRNAs generated by RDR6 via replication of viral RNAs plays a crucial role in the antiviral response of plants (Hung & Slotkin, 2021). To determine if the reduction in vsiRNAs upon importin α2 silencing corresponds to fewer secondary siRNAs, we performed an infection assay on plants with the VIGS of importin α2 in NbRDR6*i* transgenic *N*. *benthamiana* (Schwach et al., 2005). As shown in Figure 3a,b, importin α2 silencing enhanced accumulations of BaMV‐GFP, which accompanied the reduction in vsiRNAs (Figure 3b,c). We found that dual silencing of importin α2 and NbRDR6 dramatically increased the extent of BaMV accumulation, by about 2‐fold relative to importin α2*i* plants and about 1.4‐fold relative to mCherry*i*/NbRDR6*i* plants (Figure 5a,b). NbRDR6 silencing prompted a substantial reduction in accumulated levels of BaMV vsiRNAs, to approximately 10% of those observed in mCherry*i*/WT plants. Intriguingly, importin α2 and NbRDR6 dual silencing resulted in a notable 2.3‐fold increase in vsiRNAs relative to mCherry*i*/NbRDR6*i* plants (Figure 5c,d). These results support that RDR6‐dependent secondary vsiRNAs account for the reduced levels of vsiRNAs induced by importin α2 silencing and that the increase in vsiRNAs observed for importin α2 and NbRDR6 dual silenced plants might reflect primary vsiRNAs generated by robust replication of BaMV‐GFP in importin α2*i* plants.

### 2.5 NbAGO10a is upregulated by importin α2 silencing and BaMV infection

To dissect the molecular mechanism by which importin α2 silencing affects BaMV accumulation, we analysed the transcriptome of BaMV infected/uninfected mCherry*i* or importin α2*i N*. *benthamiana* plants at 5 dpi. Among approximately 500 million reads detected for each treatment, about 93% to 99% of the reads could be mapped to the viral genome and QUT database (https://sefapps02.qut.edu.au/), including BaMV mRNAs, and the genome of *N*. *benthamiana* (Table S2). By focusing on the RNA*i*‐associated genes from our RNA‐seq dataset, we found that BaMV infection induced the expression of NbAGO2, NbAGO6, NbXRN4 and NbHESO1, whereas it downregulated the expression of NbAGO1a/b, NbAGO4a/b, NbAGO7, NbRDR2, NbRDR6 and NbDCL3 (Figure S3). Importin α2 silencing curtailed the expression of NbRDR1 (Figure S3), but this protein is absent from *N*. *benthamiana* due to an insertion of in‐frame stop codons in the 5′ region (Yang et al., 2004). Notably, both NbHEN1 and NbAGO10 were upregulated upon BaMV infection of importin α2*i* plants (Figure S3).

NbHEN1 is an RNA 2′‐O‐methyltransferase that regulates the methylation of small RNAs at the 3′ terminus to prevent their turnover by innate ribonucleases (Bologna & Voinnet, 2014; Li et al., 2005; Park et al., 2002). To clarify whether the reduced levels of vsiRNAs elicited by importin α2 silencing corresponds to vsiRNA methylation, we compared the methylation status of vsiRNAs from NbHEN1*i* and importin α2*i N*. *benthamiana* by means of β‐elimination and RNA blotting. VIGS‐induced silencing of NbHEN1, which resulted in an about 60% decrease in NbHEN1 expression, did not have a profound impact on BaMV accumulation (Figure S4a,b). However, it did limit methylation of vsiRNAs, as revealed by a band shift of non‐methylated vsiRNAs after the β‐elimination treatment (arrow, Figure S4c). Conversely, importin α2 silencing did not lead to any observable shift in vsiRNAs even after β‐elimination, indicating that methylation of vsiRNAs remains unaffected in these silenced plants. Collectively, these results indicate that HEN1‐dependent methylation of vsiRNAs is not affected by the silencing of importin α2.

**FIGURE 6:**
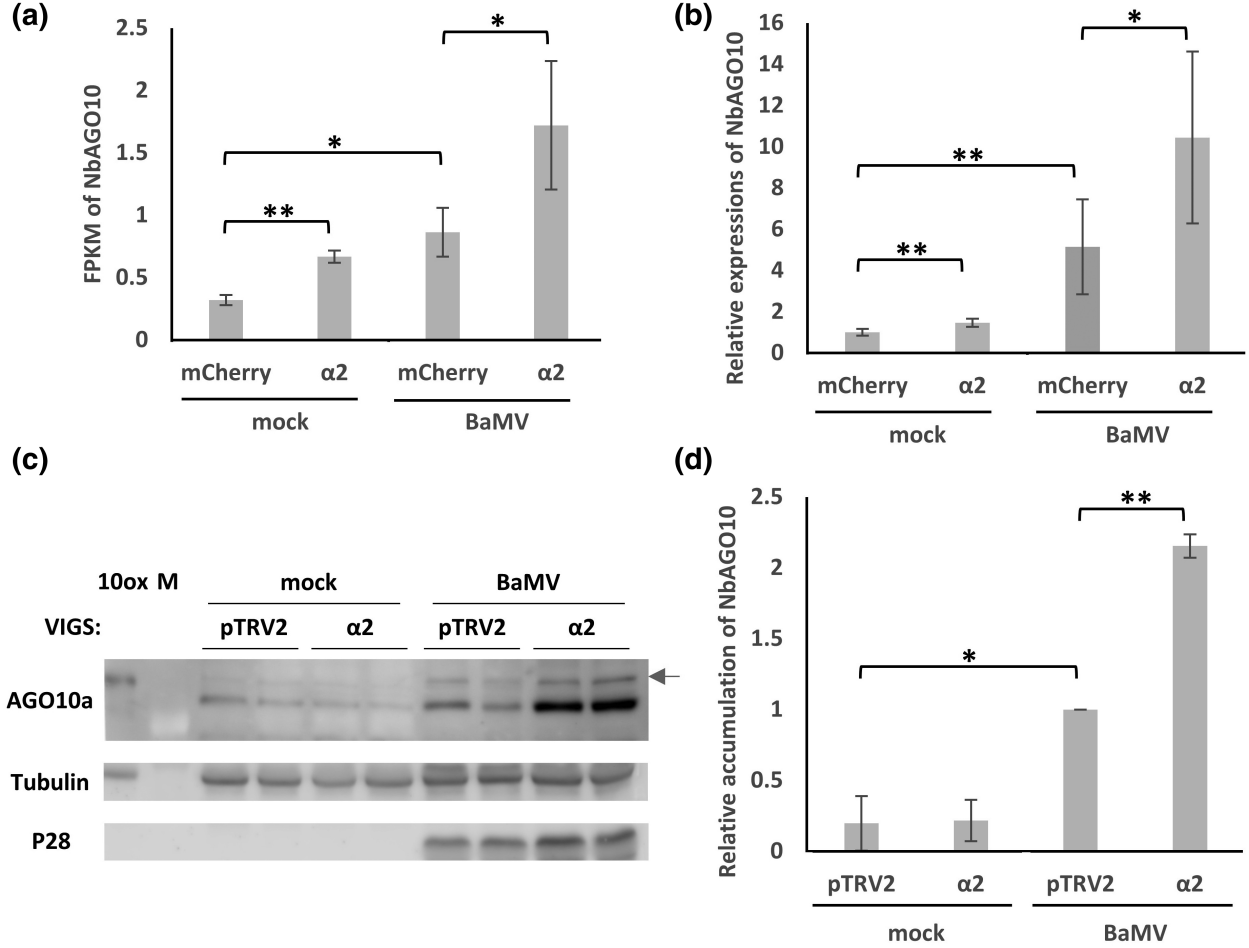
Expression and accumulation of NbAGO10 in importin α2‐silenced *Nicotiana benthamiana*. Virus‐induced gene silencing and BaMV infection (pKB) were performed as described in Figure 1. (a) Relative *NbAGO10a* expression, as determined by RNA‐seq. (b) Relative *NbAGO10a* expression, as determined by reverse transcription‐quantitative PCR (*n* = 5). (c) Accumulations of NbAGO10a and BaMV TGBp1 (P28) as determined by western blotting. NbAGO10a is indicated by blue arrow. Tubulin was used as a loading control. (d) Statistical analysis of NbAGO10 accumulation from three independent tests (*n* = 3). *p < 0.05, **p < 0.01, Student's *t* test.

NbAGO10a exerts a proviral role in terms of BaMV accumulation by sequestering vsiRNAs (Huang et al., 2019). To confirm our RNA‐seq result of upregulated *NbAGO10a* expression upon BaMV infection and importin α2 silencing (Figure 6a), we determined gene expression levels by reverse transcription‐quantitative PCR (RT‐qPCR). Consistent with our RNA‐seq data, importin α2 silencing resulted in a 1.6‐ to 2.0‐fold increase in the expression of *NbAGO10a*, regardless of BaMV infection (Figure 6b). Moreover, BaMV infection markedly enhanced *NbAGO10a* expression by 5‐fold in mCherry*i* plants, whereas it did so about 7‐fold in importin α2*i* plants (Figure 6b). Immunoblotting confirmed the enhanced NbAGO10a accumulation in BaMV‐infected and importin α2*i* plants (Figure 6c).

### 2.6 Importin α2 regulates nucleocytoplasmic shuttling of NbAGO10a in *N*. *benthamiana*

**FIGURE 7:**
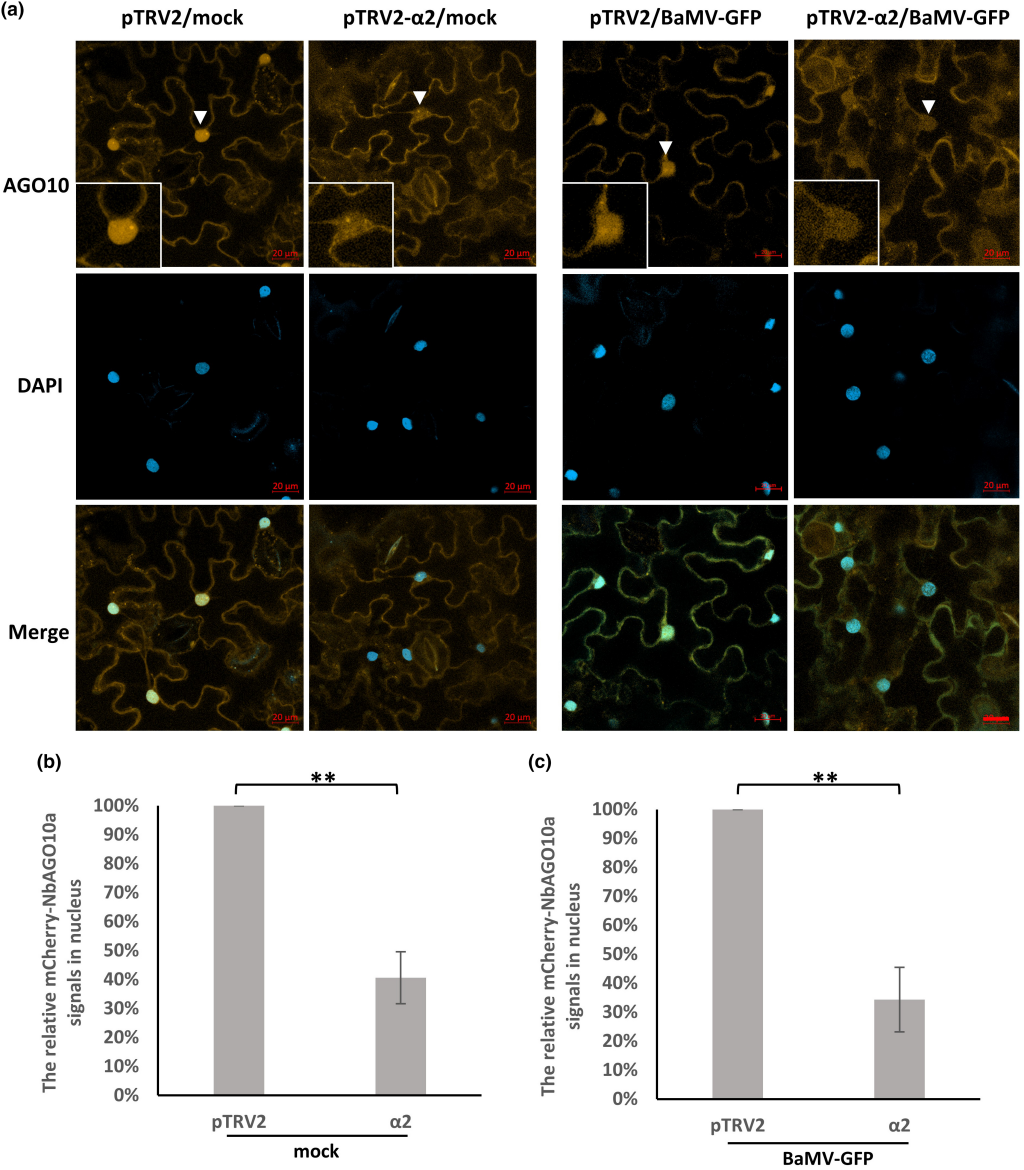
Subcellular localization of 3HA‐mCherry‐NbAGO10a in BaMV‐infected and/or importin α2‐silenced *Nicotiana benthamiana*. Virus‐induced gene silencing (VIGS) was performed as described in Figure 1. The upper leaves of silenced plants were co‐agroinfiltrated with 3HA‐mCherry‐NbAGO10a and mock (pKn) or BaMV‐GFP (pKBG) at 7 days after VIGS. (a) Localization of 3HA‐mCherry‐NbAGO10a was determined by confocal microscopy at 3 days post‐inoculation. The inserts show the nuclei indicated by arrowheads in each treatment. orange, NbAGO10a; green, BaMV‐eGFP; light blue, DAPI; bar, 20 μm. (b, c) Relative nuclear signals of 3HA‐mCherry‐NbAGO10a in mock or BaMV‐GFP infected and/or importin α2‐silenced leaves. The mean of 3HA‐mCherry‐NbAGO10a signals in the nucleus or whole cell was measured using ImageJ. The relative signals of mCherry in nuclei were calculated according to the nucleus‐to‐whole cell ratio of 3HA‐mCherry‐NbAGO10a from three independent tests (**p < 0.01, *n* = 3, Student's *t* test). Signals in pTRV2‐silenced plants were defined as 100%.

NbAGO10a harbours multiple NLS regions, specifically at amino acid positions 5–33, 59–72, 255–285, and 639–668, as predicted by NLS mapper and NLStradamus (Kosugi et al., 2009; Nguyen Ba et al., 2009). Despite these potential NLS regions, its role in regulating gene expression primarily lies at the post‐transcriptional level by interacting with small RNAs in the cytoplasm. To examine the subcellular localization of NbAGO10a, we first transiently expressed 3HA‐mCherry‐NbAGO10a in *N*. *benthamiana*. Confocal microscopy revealed that 3HA‐mCherry‐NbAGO10a was mainly located in the nucleus at 2 dpi, with moderate levels in the cytoplasm (Figure 7a). However, 7 days after importin α2 silencing, the nuclear localization of 3HA‐mCherry‐NbAGO10a had declined to about 59% relative to pTRV2‐treated wild‐type leaves of *N*. *benthamiana* (Figure 7b). Notably, the diminished nuclear presence of 3HA‐mCherry‐NbAGO10a in importin α2‐silenced plants upon BaMV infection is remarkably comparable to our observations for uninfected plants (Figure 7c). This outcome indicates that the nuclear target of 3HA‐mCherry‐NbAGO10a had been reduced in the importin α2*i* plants, irrespective of whether they had been infected by BaMV or not. Thus, importin α2 regulates the nucleocytoplasmic shuttling of NbAGO10 in *N. benthamiana*.

### 2.7 Role of the VSR TGBp1 in importin α2‐regulated BaMV accumulation

**FIGURE 8:**
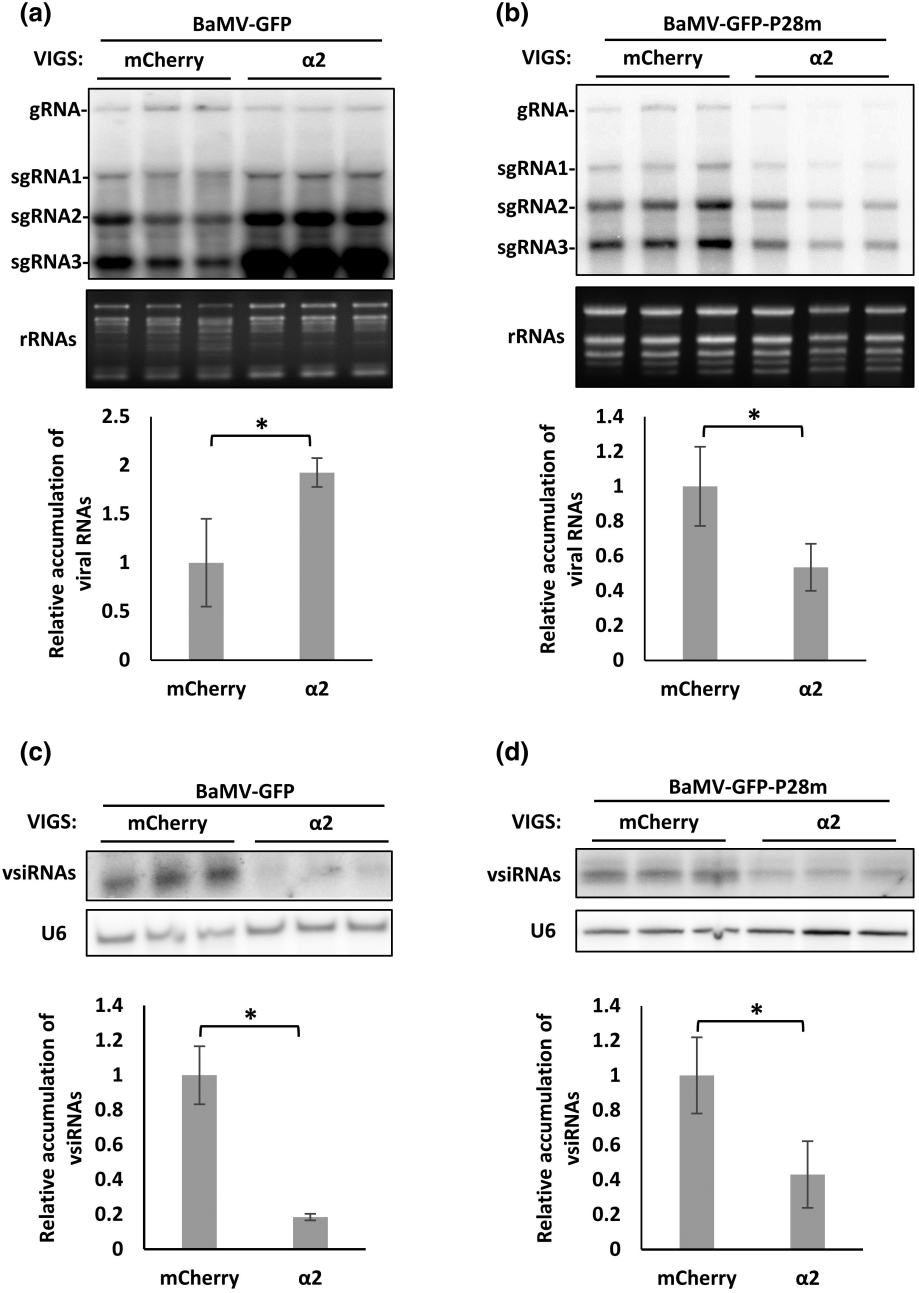
Accumulations of viral RNAs and vsiRNAs in importin α2‐silenced *Nicotiana benthamiana* infected with BaMV‐GFP or BaMV‐GFP‐P28m. Virus‐induced gene silencing (VIGS) and BaMV infection were performed as described in Figure 1. (a, b) RNA blots and statistical analysis of viral RNAs of BaMV‐GFP (a) or BaMV‐GFP‐P28m (b). (c, d) RNA blots and statistical analysis of vsiRNAs of BaMV‐GFP (c) or BaMV‐GFP‐P28m (d). Similar results were obtained from three independent tests. *p < 0.05, *n* = 3, Student's *t* test.

BaMV TGBp1 is a VSR, and it is known to interact with and upregulate NbAGO10 (Huang et al., 2019). The TGBp1 deletion mutant of BaMV‐GFP, BaMV‐GFP‐P28m, not only fails to elevate NbAGO10 expression levels, but also results in a significant reduction in viral RNA titres in *N*. *benthamiana* (Huang et al., 2019). To address if TGBp1 is involved in importin α2‐regulated BaMV accumulation, we compared vsiRNA accumulations upon infection with WT BaMV or the BaMV‐GFP‐P28m mutant in both WT and importin α2*i N*. *benthamiana* plants. Unlike the increased accumulation of BaMV‐GFP we detected upon importin α2 silencing (Figure 8a), we observed a contrasting effect for BaMV‐GFP‐P28m accumulation, which declined by about 50% (Figure 8b). Moreover, in importin α2‐silenced plants, vsiRNA accumulations declined to about 20% and about 40% those of the control plants for BaMV and BaMV‐GFP‐P28m infection treatments, respectively (Figure 8c,d). By normalizing the ratio of BaMV vsiRNAs to viral RNAs (vsiRNAs/viral RNAs), we found that the relative ratio of vsiRNAs in importin α2*i* plants was significantly reduced to 10% that of BaMV‐GFP‐infected mCherry*i* plants. Unlike for WT BaMV‐GFP, silencing of importin α2 maintained the vsiRNAs/viral RNAs ratio for BaMV‐GFP‐P28m at about 80%. These results indicate that the enhanced accumulation of BaMV triggered by importin α2 silencing had been compromised in the absence of TGBp1, supporting a critical role for TGBp1 in clearance of vsiRNAs in this regulatory mechanism.

## 3 DISCUSSION

Karyopherins are reported to be involved in various steps of RNA silencing, such as nuclear import of small RNA‐related proteins, regulation of miRNA loading into AGO proteins, nuclear export of miRNAs and transport of VSRs (Brioudes et al., 2021; Hu et al., 2021; Kim et al., 2022; Park et al., 2012; Weinmann et al., 2009; Wu et al., 2018). However, most studies have focused on importin β or exportin, with only a few investigating importin α. Herein, we demonstrate that importin α2 is also involved in regulating the RNA silencing pathway, with importin α isoforms acting as negative regulators for BaMV infection (Figures 1 and 2). The enhanced levels of BaMV accumulation in importin α2*i N*. *benthamiana* are tightly correlated to reduced levels of secondary vsiRNAs (Figure 3). Based on our findings, we postulate that importin α2 regulates the homeostasis and nucleocytoplasmic shuttling of NbAGO10a (Figures 6 and 8), which is recruited by the BaMV VSR, TGBp1 (Huang et al., 2019), to counteract the plant host antiviral response.

### 3.1 Importin α1 and α2 act as negative regulators for BaMV replication

By means of TRV‐based VIGS, we detected elevated viral titres of BaMV in the ILs of importin α1*i*, α2*i*, αc2*i*, and αc3*i N*. *benthamiana* (Figure 1c,d), as well as increased GFP signal of BaMV‐GFP in the SLs of importin α1‐ or α2‐silenced plants (Figure 1a,b). Moreover, the enhanced BaMV replication also occurs at the single cell level in importin α1‐ or α2‐silenced protoplasts (Figure 2). Together, these results indicate that importin α1 and α2 are negative regulators of BaMV replication. This scenario contrasts with previous reports stating that importin α isoforms commonly exert proviral roles during plant virus infection. For example, CaMV P6, orchid fleck virus P protein and alfalfa mosaic virus CP are transported by importin α1 or α2 to enable their nuclear accumulations and facilitate viral infection (Aparicio & Pallas, 2016; Haas et al., 2008; Kondo et al., 2013). Although both TGBp1 and CP of BaMV accumulate in the nucleus (Chang et al., 1997; Palani et al., 2009), our yeast two‐hybrid analysis reveals that TGBp1 and CP do not directly interact with importin α1 or α2 (Figure S5). Moreover, silencing of importin α1 or α2 did not alter nuclear accumulations of YFP‐TGBp1 in *N*. *benthamiana*, as determined by confocal microscopy (Figure S6a,b). In light of these findings, we suggest that, unlike other RNA viruses, nuclear import of BaMV TGBp1 and CP is not governed by importin α1 or α2. It is possible that TGBp1 and CP may passively diffuse across the nuclear membrane due to their small molecular sizes, which are below the threshold of nuclear pore complex limitations (Wang & Brattain, 2007). Thus, the importin α1/2‐independent nuclear accumulations of TGBp1 and CP prompted us to investigate the negative roles of importin α1 and α2 in BaMV infection.

Given that importin α1 and α2 share 85%–87% protein identity in their cargo‐binding domains in *N*. *benthamiana* (Conti et al., 1998), we observed similar silencing effects of importin α1 and α2 on both enhanced BaMV level and reduced vsiRNAs (Figure 3a–c), suggesting the potential partial redundancy in their roles in cellular regulatory mechanism against BaMV infection. This functional similarity is further supported by other studies, where importin α1‐ or α2‐dependent nuclear transport of cucumber mosaic virus 2b protein and *Plasmopara viticola* effector PvAVH53 facilitated the disruption of RNA silencing or innate immunity in *N*. *benthamiana*, respectively (Chen et al., 2021; Kim et al., 2022).

### 3.2 Importin α2 is involved in vsiRNA accumulation

We have demonstrated herein that silencing of importin α2 remarkably reduced BaMV vsiRNAs in *N*. *benthamiana*, as evidenced by both RNA blotting and small RNA‐seq datasets (Figures 3c and 4b–e). Expression levels of host genes associated with small RNA biogenesis and turnover, such as DCLs, AGOs (1a/b, 2, 4a/b, 5, 6, 7) and RNases (XRN2 and XRN4) all remained unchanged in the BaMV‐infected importin α2‐silenced plants (Figure S3). Even though we detected that NbHEN1 is upregulated upon importin α2 silencing following BaMV infection (Figure S3), NbHEN1 silencing did not prompt an increase in BaMV accumulation, nor did silencing of importin α2 affect 2′‐O‐methylation of vsiRNAs (Figure S4), indicating that NbHEN1‐dependent methylation of BaMV vsiRNAs is not influenced by importin α2 silencing. Moreover, our small RNA‐seq analysis revealed that both miRNAs and siRNAs in *N*. *benthamiana* were upregulated upon importin α2 silencing, independently of BaMV infection (Figure 4b–e). Taken together, these results strongly indicate that the primary processes related to biogenesis and turnover of small RNAs remains unaffected in importin α2‐silenced *N*. *benthamiana*.

RDR6 functions as an antiviral factor responsible for the synthesis of secondary siRNAs. Silencing of RDR6 increases the susceptibility of *Arabidopsis* and *N*. *benthamiana* to various viruses (Lopez‐Gomollon & Baulcombe, 2022; Qu et al., 2005; Schwach et al., 2005). Our RNA blotting analysis also showed that silencing of NbRDR6 dramatically enhanced BaMV accumulation, but it significantly reduced the levels of vsiRNAs (Figure 5), highlighting NbRDR6‐dependent synthesis of BaMV vsiRNAs. Moreover, silencing of importin α2 in NbRDR6*i N*. *benthamiana* resulted in greater accumulations of BaMV vsiRNAs than determined for mCherry*i*/NbRDR6*i* plants (Figure 5). Given that RDR6 silencing elicits a reduction in the synthesis of secondary vsiRNAs, the increased levels of BaMV vsiRNAs in the importin α2*i*/NbRDR6*i* plants must originate from the primary vsiRNAs derived from enhanced BaMV accumulation. Thus, the reduction in vsiRNAs attributable to importin α2 silencing affects the extent of secondary vsiRNAs generated by the activity of NbRDR6.

### 3.3 Importin α2 regulates TGBp1‐NbAGO10a‐SDN1‐mediated clearance of vsiRNAs

In addition to RDR6, NbAGO10 is also known to compete with NbAGO1 for binding to the BaMV vsiRNAs degraded by NbAGO10‐recruited SDN1 exonuclease, thereby facilitating BaMV infection (Huang et al., 2019). This mechanism is similar to *Arabidopsis* AGO10, which facilitates the turnover of miR155/156 via SDN1 and SDN2 exonucleases (Yu et al., 2017). Results from our RNA‐seq, RT‐qPCR and immunoblotting experiments clearly demonstrate upregulation of NbAGO10a expression, at both RNA and protein levels, in importin α2‐silenced plants following BaMV infection (Figures 6 and S3). Moreover, BaMV TGBp1 can promote NbAGO10a expression and recruit NbAGO10 and SDN1 exonuclease to accelerate turnover of BaMV vsiRNAs in *N*. *benthamiana* (Huang et al., 2019). The TGBp1 deletion mutant, BaMV‐GFP‐P28m, suppressed this enhancement of BaMV accumulation arising from the elevated ratio of vsiRNAs‐to‐BaMV RNAs in importin α2‐silenced *N*. *benthamiana* (Figure 8), indicating that TGBp1 is indispensable for importin α2*i*‐mediated BaMV enhancement. BaMV TGBp1 is a multifunctional protein. Notably, it exhibits VSR activity to enhance BaMV accumulation (Huang et al., 2019). Additionally, it holds RNA binding activity and interacts with TGBp2 and CP, thereby initiating the assembly of BaMV RNP involving viral RNAs, and TGBp3 at ER or Golgi membrane for BaMV movement (Chou et al., 2013; Huang et al., 2020; Wung et al., 1999). Moreover, it interacts with mitochondria voltage‐dependent anion channel proteins, playing a crucial role in inducing dynamic BaMV replication complex (Lee et al., 2022). Consequently, the presence of TGBp1 within the dynamic BaMV‐induced replication complex or RNP movement complex becomes pivotal, enabling BaMV to evade RNA silencing responses.

We found that NbAGO10a, which contains multiple putative NLS regions, predominantly localized in the nucleus of WT *N. benthamiana* (Figure 7a), so its nuclear import may rely on an importin α/β‐dependent pathway. Nevertheless, suppression of importin α2 resulted in significantly increased accumulations of 3HA‐mCherry‐NbAGO10a within the cytoplasm (Figure 7), where both RNA virus replication and post‐transcriptional gene silencing take place. Hence, the silencing of importin α2 that triggers elevated NbAGO10a expression in the cytoplasm may foster BaMV accumulations via TGBp1‐NbAGO10a‐SDN1‐mediated clearance of vsiRNAs. However, this increase in viral RNAs and reduction of vsiRNAs were not observed in importin α1‐ or α2‐silenced plants infected with another potexvirus, PVX (Figure 3d,e). The discrepancy in viral accumulation between BaMV and PVX may be due to differences between them in infection and replication strategies. Notably, whereas overexpression of NbAGO10a can enhance BaMV titres, it does not promote increased levels of PVX accumulation in *N*. *benthamiana* (Huang et al., 2019). Moreover, PVX replication takes place within the cytoplasmic X‐body (Tilsner et al., 2012), whereas BaMV replication is tightly associated with chloroplasts (Cheng et al., 2013) and mitochondria (Lee et al., 2022). The mechanisms underlying these differential findings for these two potexviruses warrant further investigation.

## 4 EXPERIMENTAL PROCEDURES

### 4.1 Plasmids

For VIGS, partial sequences of NbHEN1 and importin α isoforms were cloned from the cDNA of *N*. *benthamiana* by PCR using specific primers (Table S3) and fused into pTRV2 (Dinesh‐Kumar et al., 2003) with specific restriction enzymes (Table S3). The same strategy was used to generate pTRV2‐mCherry using pBin‐mCherry‐NbFIB2 plasmid (Chang et al., 2016) as the template for PCR amplification (Table S3). For yeast two‐hybrid analysis, importin α1, importin α2, CP, and TGBp1 were fused with pAD‐GAL4‐2.1 or pBD‐GAL4 Cam plasmids (Agilent Tech. Inc.) using specific primers and restriction enzymes (Table S3). The pCB plasmid, an infectious clone of BaMV (Lin et al., 2004), was used to examine BaMV accumulation in *N. benthamiana* protoplasts. For transient expression, pBA‐Y‐p1(YFP‐TGBp1) was obtained from the laboratory of Ban‐Yang Chang (Chou et al., 2013). The pBin‐P20‐eGFP, pKn, pKB, pKBG and pKnPVX plasmids have been described in previous reports (Chang et al., 2016; Huang et al., 2019; Liou et al., 2014; Prasanth et al., 2011). To generate pBIN61‐3HA‐NbAGO10a‐mCherry, NbAGO10a was PCR‐amplified using specific primers containing restriction enzyme sites (Table S3) and introduced into pBIN61 plasmid (Bendahmane et al., 2000) containing three HA tags and mCherry fluorescence protein.

### 4.2 VIGS, virus infection, RNA extraction and RNA blotting

*Agrobacterium tumefaciens* C58C1 was used for VIGS or agroinfiltration according to previous reports (Chang et al., 2016; Liou et al., 2014). We used 18‐day‐old WT or NbRDR6*i* transgenic *N*. *benthamiana* (Schwach et al., 2005) for VIGS by agroinfiltration using *A. tumefaciens* C58C1 harbouring the indicated pTRV1, pTRV2‐mCherry, pTRV2‐imp α1, pTRV2‐imp α2, pTRV2‐imp αc2, pTRV2‐imp αc3 or pTRV2‐hen1 plasmid. At 7 days after VIGS, the upper leaves of silenced plants were agroinfiltrated with agrobacteria harbouring pKB, pKBG or pKnPVX. ILs were sampled at 5 days post‐infiltration (dpi), and SLs were harvested at 14 dpi. Total RNA of *N*. *benthamiana* leaves was purified using TRIzol (Invitrogen) according to the manufacturer's instructions. RNA blotting was performed as described previously (Chang et al., 2016). The BaMV‐ and PVX‐specific probes were generated from HindIII‐linearized pBaHB or pPVXHE plasmid according to previous reports (Huang et al., 2012; Lin et al., 1993). Small RNA blotting and probe synthesis were conducted as described previously (Lin et al., 2010).

### 4.3 Protein extraction and immunoblotting

Total protein isolation and immunoblotting were performed according to previous reports (Chou et al., 2013; Lukhovitskaya et al., 2015). Rabbit anti‐tubulin (AS10 680; Agrisera) and mouse anti‐actin (A0480; Sigma‐Aldrich) were used as the internal control. The anti‐AGO10a, anti‐CP and anti‐P28 antibodies were kindly provided by Yau‐Heiu Hsu (Huang et al., 2019).

### 4.4 Protoplast transfection

The leaves of *N*. *benthamiana* were harvested for protoplast isolation 9 days after VIGS. Protoplast isolation and transfection were performed following the previous reports (Cheng et al., 2009; Dai & Wang, 2022; Sheen, 2001). Briefly, about 10^5^ protoplasts were incubated with 1 μg pCB in MMG buffer (15 mM MgCl_2_, 0.55 M mannitol‐MES, pH 5.7) for transfection. An equal volume of 40% PEG 4000 was added into the mixture and incubated at room temperature for 30 min. The transfected protoplasts were washed with 1 mL MMC buffer (20 mM CaCl_2_, 0.55 M mannitol‐MES, pH 5.7) twice and then incubated in growth buffer (1 μM CuSO_4_, 1 μM KI, 1 mM MgSO_4_, 0.2 mM K_2_HPO_4_, 1 mM KNO_3_, 10 mM CaCl_2_, 0.55 M mannitol‐MES, pH 5.7) at room temperature under constant light for 20 h.

### 4.5 Leaf GFP image assay

The GFP fluorescence of BaMV‐GFP‐infected leaves was detected and quantified using an IVIS Lumina III LT In Vivo Imaging System (Perkin Elmer) and software (Xenogen). All experiments were conducted in triplicate.

### 4.6 RT‐qPCR

*N*. *benthamiana* cDNA was synthesized using a TOOLS Easy Fast RT Kit (Biotools) according to the manufacturer's instructions. qPCR was performed using SYBR Green PCR Master Mix (Applied Biosystems) with an Applied Biosystems QuantStudio 12 K Flex Real‐Time PCR system (ThermoFisher Scientific). The primers used for RT‐qPCR are listed in Table S3.

### 4.7 Transient expression and confocal microscopy

The upper leaves of silenced plants were induced to transiently express target proteins by agroinfiltration of the indicated plasmids at 10 days after VIGS. Plant leaves were stained with DAPI (1 μg/mL) as a nuclear marker 30 min before microscopy. Confocal images were captured using an LSM 510 META confocal microscope or an LSM880 Airyscan confocal microscope (Zeiss). The eGFP, YFP, mCherry and DAPI fluorescence signals were detected by excitation lasers at 488, 514, 561 and 405 nm, and emission filters at 505–550 nm, 515–560 nm, 573–629 nm and 420–480 nm, respectively. Signal intensities and image area were measured in ImageJ (https://imagej.net/ij/index.html).

### 4.8 β‐elimination of small RNAs

To assess methylation of vsiRNAs, we enriched for small RNAs from total RNAs using a mirVANA miRNA Isolation Kit (Invitrogen) according to the user's manual. β‐elimination of small RNAs was performed according to the protocol of Yang et al. (2007) with some modifications. In brief, small RNAs were incubated in borax/boric acid buffer (0.06 M, pH 8.6) with 25 mM sodium periodate in the dark at room temperature for 1 h. Then, the reaction was stopped by adding 1/10 volume of glycerol and incubated in the dark at room temperature for 30 min. The small RNAs were precipitated with ethanol and incubated in borax/boric acid buffer (0.06 M, pH 9.5) at 45°C for 90 min. The small RNAs were then precipitated again with ethanol, separated by 20% PAGE with 7 M urea, and detected by small RNA blotting.

### 4.9 Yeast two‐hybrid assay

Yeast transformation and yeast two‐hybrid analysis were performed according to the HybriZAP‐2.1 two‐hybrid libraries instruction manual (Agilent). Interactions were verified at 3 days after incubation. A combination of AD plasmid expressing SV40 large T antigen and BD plasmid with P53 tumour suppressor was used as a positive control. A combination of SV40 large T antigen and human lamin C was used as the negative control.

### 4.10 Library preparation for RNA‐seq

Libraries for RNA‐seq were prepared by following the manufacturer's protocol for the Illumina TruSeq Stranded mRNA Sample Preparation Kit (RS‐122‐2103, Illumina). In brief, 3 μg of total RNA was used for library construction. Poly(A) RNA was captured by oligo‐dT beads and fragmented upon elution from the beads. The first‐strand cDNA was synthesized by SuperScript III reverse transcriptase (Invitrogen) using dNTPs and random primers. The second‐strand cDNA was generated using a dUTP mix. A single A base was added to the 3′ end of the double‐stranded (ds) cDNA, before ligating barcoded Truseq adapters. The resulting products were purified and enriched by means of 12 cycles of PCR to create the final double‐stranded cDNA library. A final size selection was performed by means of 1.4% low‐range agarose (Bio‐Rad) gel electrophoresis to yield a library of inserts 200–400 bases in length. The library was extracted from the agarose gel using a MinElute PCR Purification Kit (Qiagen). Final libraries were analysed using a Fragment Analyser HS NGS Fragment Kit (Agilent) to estimate quantity and check size distribution. The prepared libraries were pooled and subjected to Illumina HiSeq 4000 sequencing.

### 4.11 RNA‐seq read mapping

The qualified RNA‐seq reads were filtered using AFTERQC (Chen et al., 2017) to remove Illumina adaptor sequences and low‐quality reads and applying a Phred33 quality score cut‐off of 20 and minimum read length of 35 bp. Pre‐ and post‐filter quality statistics for each sample were calculated in FastQC (Andrews, 2015). Reads mapping to the annotated *N*. *benthamiana* proteome (Kourelis et al., 2019) were obtained by running ‘bowtie2’ (Langmead & Salzberg, 2012) with default parameters on reads for each sample. The SAMtools program (Li et al., 2009) was used to sort bam files and calculate alignment statistics. R/Bioconductor package DESeq2 (Love et al., 2014) was used to calculate the normalized expression levels of different samples and to merge the data into a FPKM (fragments per kilobase transcriptome per million fragments) matrix for further analysis.

### 4.12 Small RNA‐seq

Small RNA libraries were prepared from total RNA by TruSeq small RNA Sample Prep Kit (Illumina) for deep sequencing on an Illumina HiSeq‐2500 platform according to the manufacturer's instructions. FastQC (Illumina) was used for quality control. Adaptor trimming was performed in Cutadapt (https://cutadapt.readthedocs.io/en/stable/). Trimmed reads of 19–25 nucleotides were considered for further analysis. The small RNA reads were mapped to BaMV sequences using SearchSmallRNA (http://www.microbiologia.ufrj.br/ssrna/). Conserved and premature miRNAs, structural RNAs, and *N*. *benthamiana* genome sequences were identified by submitting the processed siRNA reads to the standalone SRNABench platform (https://bioinfo2.ugr.es/ceUGR/srnabench/). The database of *N*. *benthamiana* miRNAs was obtained from the QUT *N*. *benthamiana* genome draft (draft genome assembly v0.5, http://benthgenome.qut.edu.au/). The database of *N*. *benthamiana* structural RNAs was taken from Rfam (https://rfam.xfam.org/). miRNA‐related small RNAs were searched against the annotated proteome of *N*. *benthamiana* (Kourelis et al., 2019).

## Supporting information


**Figure S1.** Phenotypes and expression patterns of importin α isoforms in silenced *Nicotiana benthamiana*. Virus‐induced gene silencing (VIGS) was performed as described in Figure 1. (a) Phylodendrogram of importin α isoforms in *N*. *benthamiana*. The numbers indicate the accession numbers from Sol genomics database or GenBank. Asterisks show the targets of VIGS in the experiments. (b) The phenotypes of *N*. *benthamiana* 13 days after silencing of importin α isoforms. (c–f) Reverse transcription‐quantitative PCR analyses of importin α1 (c), α2 (d), αc2 (e), and αc3 (f) after VIGS of indicated plants at 13 days post‐agroinfiltration. *Actin* was used as an internal control. Significant differences, *p < 0.05, **p < 0.01, ***p < 0.001, *n* = 3, Student’s *t* test.

**Figure S2.** Alignment of vsiRNA reads to the BaMV genome. (a) Map of the BaMV genome. The relative positions of RdRp, TGBp1‐3, and CP in the genome are indicated by arrows. (b, c) The distribution of 21 nucleotide (nt) (b) and 22 nt (c) vsiRNAs of BaMV in mCherry‐ or importin α2‐silenced leaves. The positive or negative RPM indicates the abundance of positive‐ or negative‐stranded BaMV RNAs, respectively. The *x* axis indicates the position of vsiRNAs in the BaMV genome.

**Figure S3.** Expression patterns of RNA silencing‐associated genes in BaMV‐infected and mCherry‐ or importin α2‐silenced *Nicotiana benthamiana*. Virus‐induced gene silencing and BaMV infection (pKB) were performed as described in Figure 1. The infected leaves were sampled at 5 days post‐inoculation for RNA‐seq. After trimming, gene reads were identified by aligning them against the QUT *N. benthamiana* Genome & Transcriptome database. DRB, double‐stranded RNA‐binding protein; RDR, RNA‐dependent RNA polymerase; SDN4, putative small RNA degrading nuclease 4; NRPD, DNA‐directed RNA polymerase IV subunit; 4; XRN, 5′‐3′ exoribonuclease; HESO1, HEN1 suppressor 1; URT1, UTP:RNA uridylyltransferase 1; SN4TDR, staphylococcal nuclease domain‐containing protein 1‐like; AIN1, 5′‐3′ exoribonuclease 4; CMT3a/b, chromomethylase 3a/b; MET1, methyltransferase 1; HST1, Hasty 1 (a transporter).

**Figure S4.** HEN1‐dependent 2′‐O‐methylation of BaMV vsiRNAs is not affected by importin α2 silencing. (a, b) Reverse transcription‐quantitative PCR of *NbHEN1* (a) and BaMV (b) in importin α2*i* or NbHEN1*i N Nicotiana benthamiana* leaves. The procedures for VIGS and BaMV infection (pKB) are described in Figure 1. *Actin* was used as the internal control (*p < 0.05, **p < 0.01, *n* = 4, Student’s *t* test). (c) The 21‐nucleotide synthetic siRNA (left) or total small RNAs (right) were treated with (+) or without (−) β‐elimination and detected by RNA blots. Arrow indicates the band shift of siRNAs after β‐elimination. C, mock plant.

**Figure S5.** No interaction of importin α isoforms with BaMV TGBp1 or CP, as determined by yeast two‐hybrid analysis. Dilutions of yeast droplets and the synthetic dropout medium (SD) without Trp/Leu/His or Trp/Leu are indicated. Positive control, pAD‐SV40/pBD‐P53; negative control, pAD‐SV40/pBD‐lamin C.

**Figure S6.** Relative nuclear accumulations of YFP‐TGBp1 in mCherry‐, importin α1‐ or importin α2‐silenced *Nicotiana benthamiana*. Virus‐induced gene silencing (VIGS) was performed as described in Figure 1. YFP‐TGBp1 was expressed by agroinfiltration at 7 days after VIGS and detected at 2 days post‐agroinfiltration by confocal microscopy. (a) The intracellular localization of YFP‐TGBp1. DAPI was used as the nuclear marker. Scale bar: 20 μm. (b) The nuclear accumulation of YFP‐TGBp1 after VIGS. The mean of YFP‐TGBp1 signal in the nucleus or whole cell was measured using ImageJ. The YFP signal in the nucleus was calculated according to the nucleus‐to‐whole cell ratio of YFP‐TGBp1 (*n* = 6, Student’s *t* test).

**Table S1.** Summary of small RNA‐seq and mapping results.

**Table S2.** Summary of RNA‐seq and mapping results.

**Table S3.** List of primers in the experiments.

## Data Availability

The data that support the findings of this study are available in NCBI Sequence Read Archive (SRA) at https://www.ncbi.nlm.nih.gov/. The RNA‐seq data are available from the following accessions: SAMN36706531, SAMN36706532, SAMN36706533, SAMN36706534, SAMN36706535, SAMN36706536, SAMN36706537, SAMN36706538, SAMN36706539, SAMN36706540, SAMN36706541, SAMN36706542, SAMN36706543, SAMN36706544, SAMN36706545, SAMN36706546, SAMN36706547, SAMN36706548, SAMN36706549, SAMN36706550, SAMN36706551, SAMN36706552, SAMN36706553, and SAMN36706554. The small RNA‐seq data is available from the following accessions: SAMN37810561, SAMN37810562, SAMN37810563, SAMN37810564, SAMN37810565, SAMN37810566, SAMN37810567, SAMN37810568, SAMN37810569, SAMN37810570, SAMN37810571, SAMN37810572, SAMN37810573, SAMN37810574, SAMN37810575, SAMN37810576, SAMN37810577, SAMN37810578, SAMN37810579, SAMN37810580, SAMN37810581, SAMN37810582, SAMN37810583, SAMN37810584, SAMN37810585, SAMN37810586, SAMN37810587, SAMN37810588, SAMN37810589, SAMN37810590, SAMN37810591, SAMN37810592, SAMN37810593, SAMN37810594, SAMN37810595, and SAMN37810596.
