# Supplementary figures and images for: Importin α2 participates in RNA interference against bamboo mosaic virus accumulation in Nicotiana benthamiana via NbAGO10a‐mediated small RNA clearance
Source: Mol Plant Pathol. 2024 Jan 19;25(1):e13422. doi: 10.1111/mpp.13422 (PMC10799208; doi:10.1111/mpp.13422)

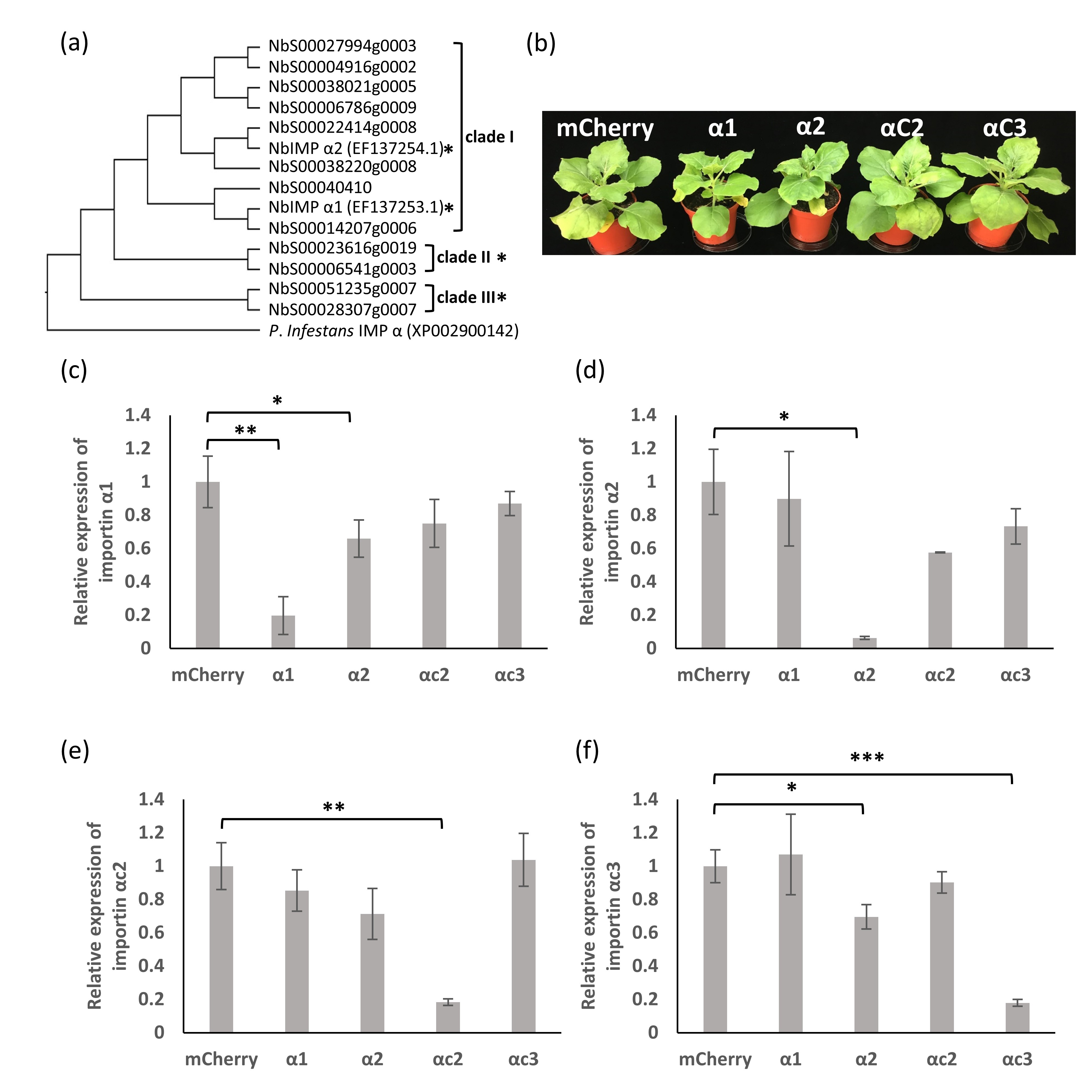

Supplement: Supplementary file 1 — Figure S1. Phenotypes and expression patterns of importin α isoforms in silenced Nicotiana benthamiana. Virus‐induced gene silencing (VIGS) was performed as described in Figure 1. (a) Phylodendrogram of importin α isoforms in N. benthamiana. The numbers indicate the accession numbers from Sol genomics database or GenBank. Asterisks show the targets of VIGS in the experiments. (b) The phenotypes of N. benthamiana 13 days after silencing of importin α isoforms. (c–f) Reverse transcription‐quantitative PCR analyses of importin α1 (c), α2 (d), αc2 (e), and αc3 (f) after VIGS of indicated plants at 13 days post‐agroinfiltration. Actin was used as an internal control. Significant differences, *p < 0.05, **p < 0.01, ***p < 0.001, n = 3, Student’s t test. [file MPP-25-e13422-s003.jpg]

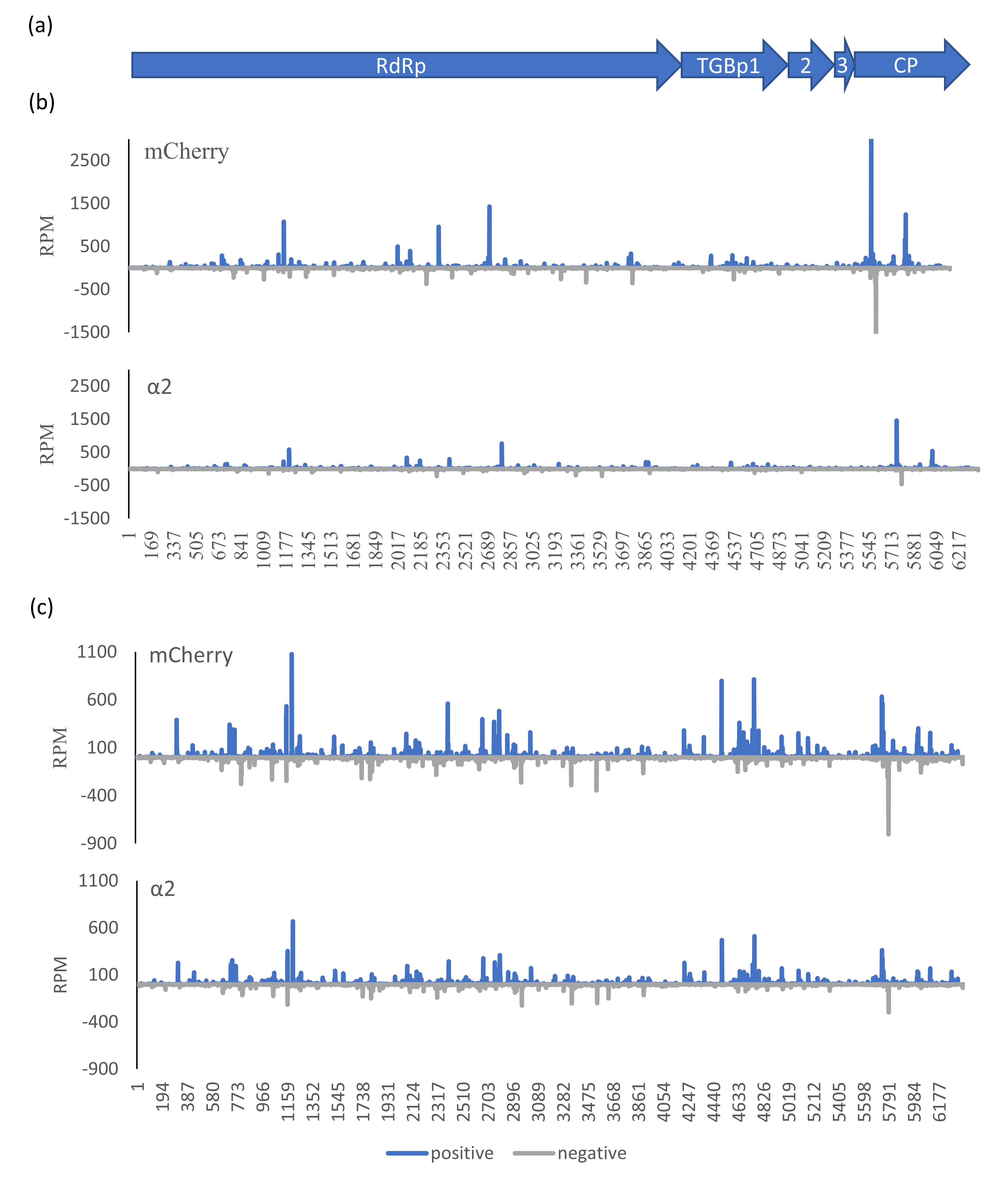

Supplement: Supplementary file 2 — Figure S2. Alignment of vsiRNA reads to the BaMV genome. (a) Map of the BaMV genome. The relative positions of RdRp, TGBp1‐3, and CP in the genome are indicated by arrows. (b, c) The distribution of 21 nucleotide (nt) (b) and 22 nt (c) vsiRNAs of BaMV in mCherry‐ or importin α2‐silenced leaves. The positive or negative RPM indicates the abundance of positive‐ or negative‐stranded BaMV RNAs, respectively. The x axis indicates the position of vsiRNAs in the BaMV genome. [file MPP-25-e13422-s002.jpg]

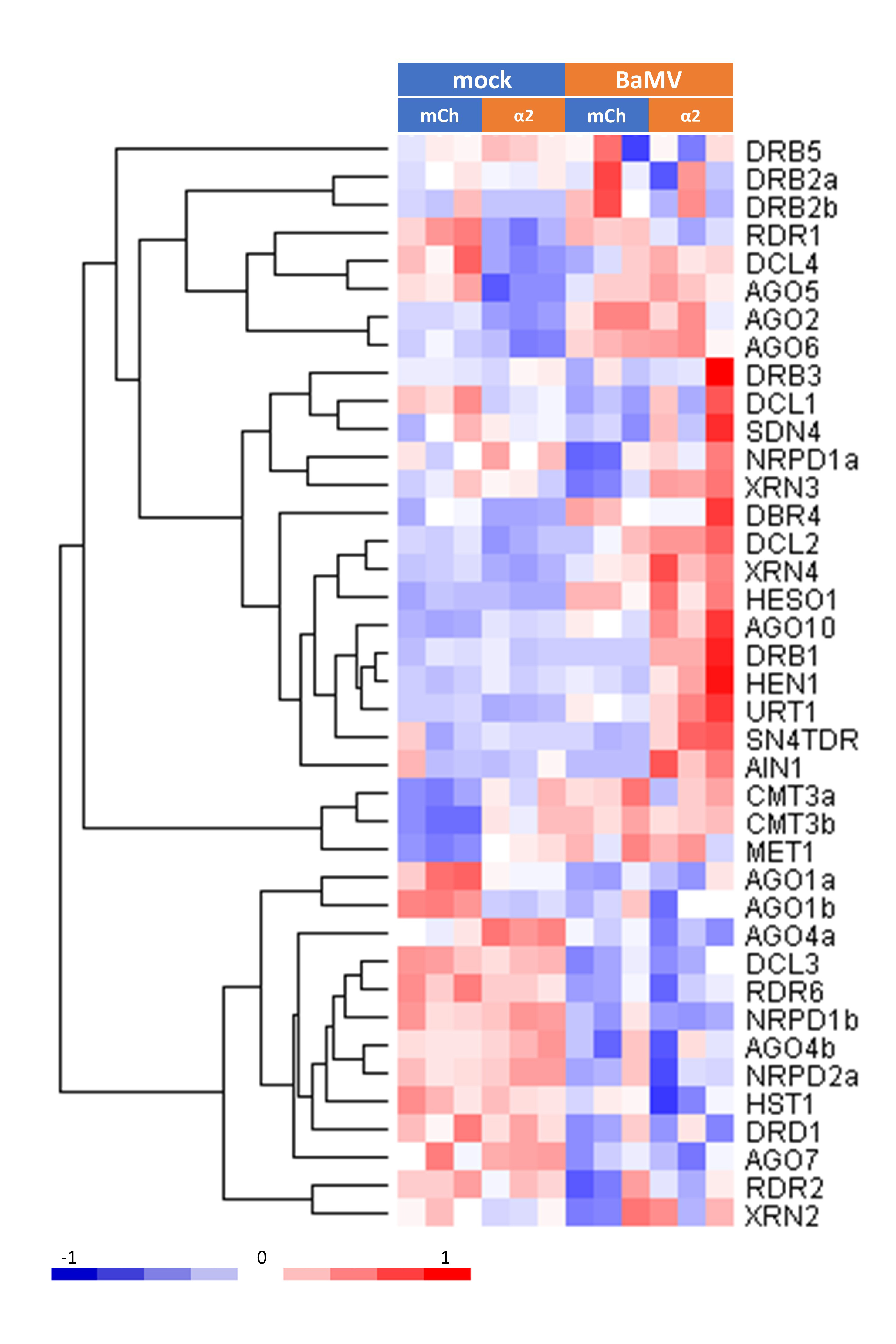

Supplement: Supplementary file 3 — Figure S3. Expression patterns of RNA silencing‐associated genes in BaMV‐infected and mCherry‐ or importin α2‐silenced Nicotiana benthamiana. Virus‐induced gene silencing and BaMV infection (pKB) were performed as described in Figure 1. The infected leaves were sampled at 5 days post‐inoculation for RNA‐seq. After trimming, gene reads were identified by aligning them against the QUT N. benthamiana Genome & Transcriptome database. DRB, double‐stranded RNA‐binding protein; RDR, RNA‐dependent RNA polymerase; SDN4, putative small RNA degrading nuclease 4; NRPD, DNA‐directed RNA polymerase IV subunit; 4; XRN, 5′‐3′ exoribonuclease; HESO1, HEN1 suppressor 1; URT1, UTP:RNA uridylyltransferase 1; SN4TDR, staphylococcal nuclease domain‐containing protein 1‐like; AIN1, 5′‐3′ exoribonuclease 4; CMT3a/b, chromomethylase 3a/b; MET1, methyltransferase 1; HST1, Hasty 1 (a transporter). [file MPP-25-e13422-s007.jpg]

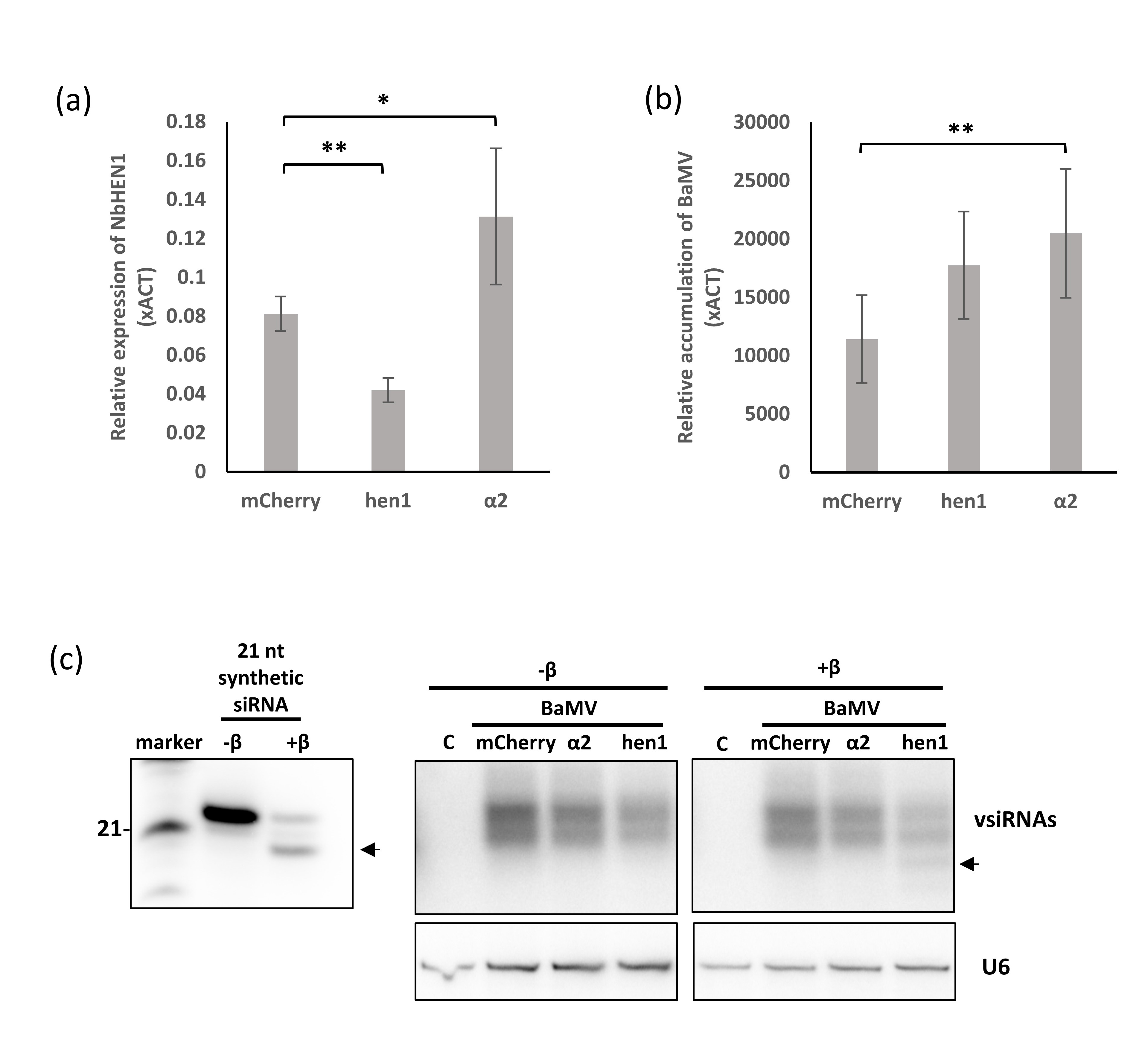

Supplement: Supplementary file 4 — Figure S4. HEN1‐dependent 2′‐O‐methylation of BaMV vsiRNAs is not affected by importin α2 silencing. (a, b) Reverse transcription‐quantitative PCR of NbHEN1 (a) and BaMV (b) in importin α2i or NbHEN1i N Nicotiana benthamiana leaves. The procedures for VIGS and BaMV infection (pKB) are described in Figure 1. Actin was used as the internal control (*p < 0.05, **p < 0.01, n = 4, Student’s t test). (c) The 21‐nucleotide synthetic siRNA (left) or total small RNAs (right) were treated with (+) or without (−) β‐elimination and detected by RNA blots. Arrow indicates the band shift of siRNAs after β‐elimination. C, mock plant. [file MPP-25-e13422-s008.jpg]

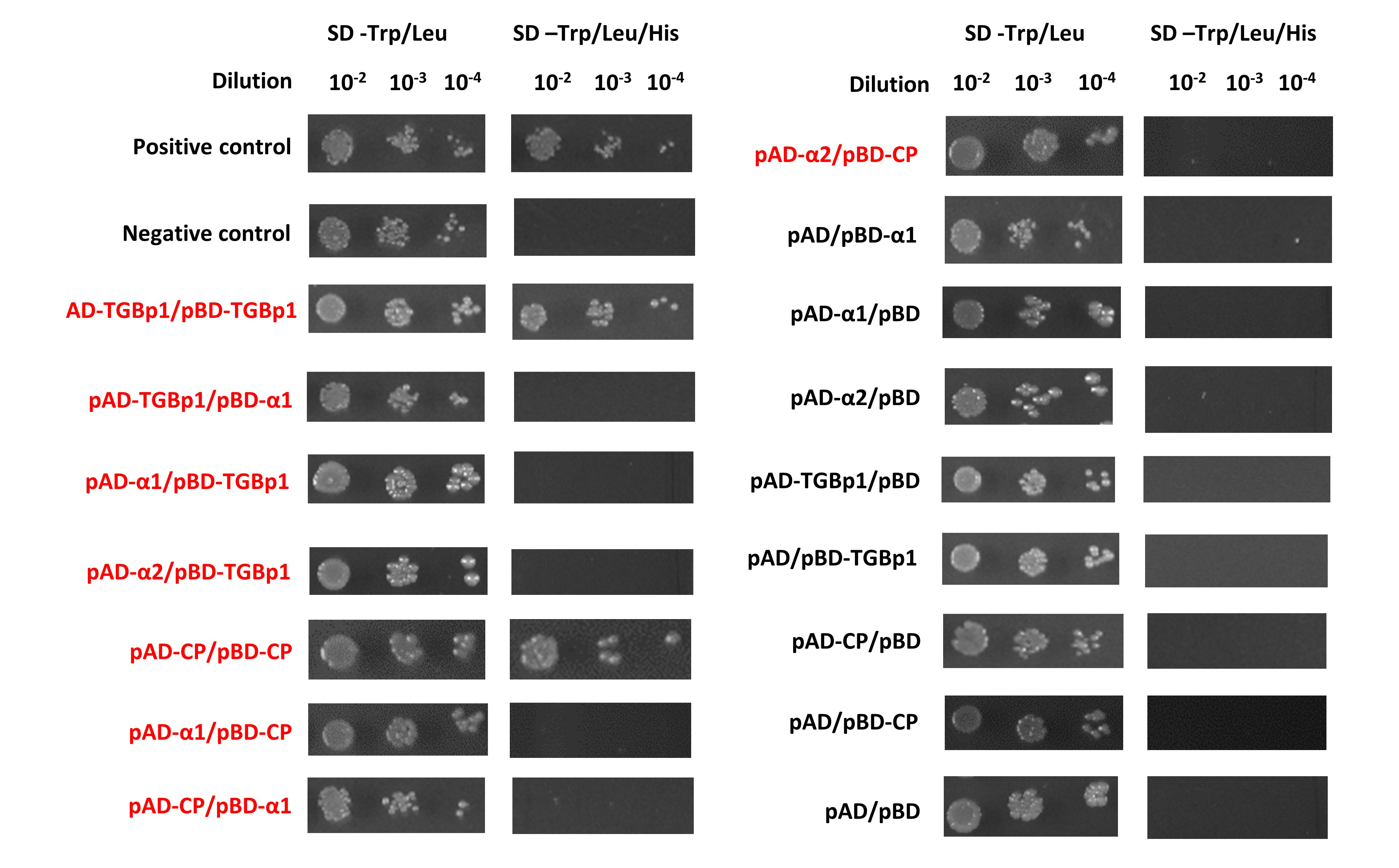

Supplement: Supplementary file 5 — Figure S5. No interaction of importin α isoforms with BaMV TGBp1 or CP, as determined by yeast two‐hybrid analysis. Dilutions of yeast droplets and the synthetic dropout medium (SD) without Trp/Leu/His or Trp/Leu are indicated. Positive control, pAD‐SV40/pBD‐P53; negative control, pAD‐SV40/pBD‐lamin C. [file MPP-25-e13422-s005.jpg]

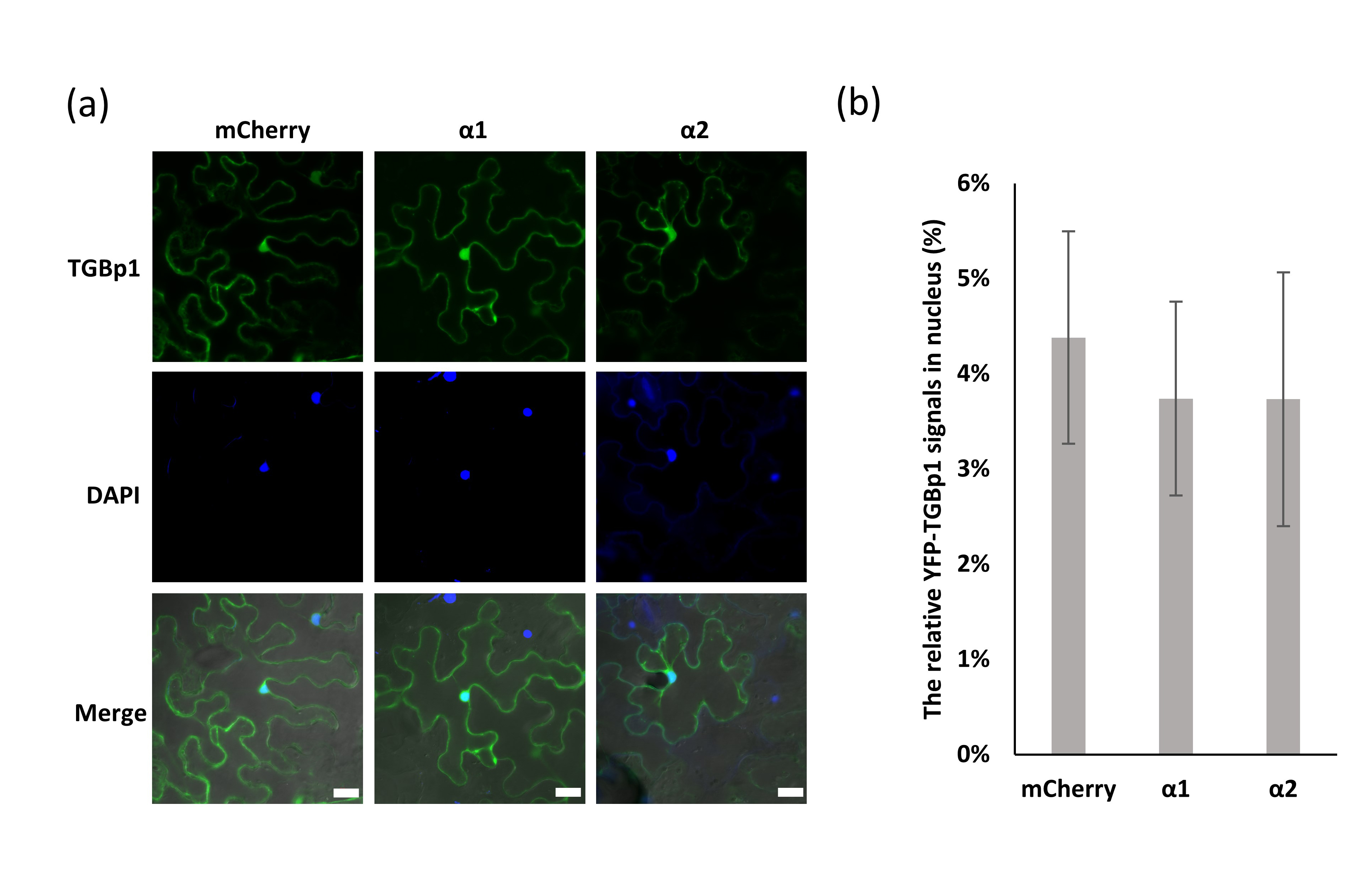

Supplement: Supplementary file 6 — Figure S6. Relative nuclear accumulations of YFP‐TGBp1 in mCherry‐, importin α1‐ or importin α2‐silenced Nicotiana benthamiana. Virus‐induced gene silencing (VIGS) was performed as described in Figure 1. YFP‐TGBp1 was expressed by agroinfiltration at 7 days after VIGS and detected at 2 days post‐agroinfiltration by confocal microscopy. (a) The intracellular localization of YFP‐TGBp1. DAPI was used as the nuclear marker. Scale bar: 20 μm. (b) The nuclear accumulation of YFP‐TGBp1 after VIGS. The mean of YFP‐TGBp1 signal in the nucleus or whole cell was measured using ImageJ. The YFP signal in the nucleus was calculated according to the nucleus‐to‐whole cell ratio of YFP‐TGBp1 (n = 6, Student’s t test). [file MPP-25-e13422-s004.jpg]
