# Supplementary material for: Importin α2 participates in RNA interference against bamboo mosaic virus accumulation in Nicotiana benthamiana via NbAGO10a‐mediated small RNA clearance
Source: Mol Plant Pathol. 2024 Jan 19;25(1):e13422. doi: 10.1111/mpp.13422 (PMC10799208; doi:10.1111/mpp.13422)
Supplement: Supplementary file 7 — Table S1. Summary of small RNA‐seq and mapping results. [file MPP-25-e13422-s001.docx]

Table S1. Summary of small RNA-seq and mapping results

|  | **mock + mCherry*i*** | **mock + imp α2*i*** | **BaMV + mCherry*i*** | **BaMV + imp α2*i*** |
| --- | --- | --- | --- | --- |
| **Raw reads** | 136,796,318 | 144,336,918 | 145,754,794 | 138,920,558 |
| **High quality reads*** | 71,989,535 | 69,584,414 | 111,572,145 | 102,449,571 |
| **BaMV vsiRNAs*** | 19,448 | 24,324 | 26,355,960 (24%) | 15,382,428 (15%)^+^ |
| **miRNAs*** | 1,641,286 (2%)^+^ | 2,207,538 (3%)^+^ | 2,699,147 (2%)^+^ | 3,990,787 (4%)^+^ |
| **mRNA-derived small RNAs*** | 10,577,518 (15%)^+^ | 12,784,625 (18%)^+^ | 19,607,669 (18%)^+^ | 22,957,147 (22%)^+^ |
| **Structural RNAs*** | 4,193,272 (6%)^+^ | 5,829,671 (8%)^+^ | 2,118,839 (2%)^+^ | 2,649,746 (3%)^+^ |

* Reads from 19-25 nts

+ brackets indicate the relative ratio compared to total reads (19-25 nts)
