# Supplementary material for: Importin α2 participates in RNA interference against bamboo mosaic virus accumulation in Nicotiana benthamiana via NbAGO10a‐mediated small RNA clearance
Source: Mol Plant Pathol. 2024 Jan 19;25(1):e13422. doi: 10.1111/mpp.13422 (PMC10799208; doi:10.1111/mpp.13422)
Supplement: Supplementary file 8 — Table S2. Summary of RNA‐seq and mapping results. [file MPP-25-e13422-s006.docx]

Table S2 Summary of RNA-seq and mapping results

|  | **Total clean reads** | **mRNAs of *N*. *benthamiana*** | **Genome of *N*. *benthamiana*** | **BaMV** |
| --- | --- | --- | --- | --- |
| **mock + mCherry*i*** | 497,055,257 | 38.0% | 55.4% | 0.2% |
| **mock + imp α2*i*** | 452,096,792 | 37.9% | 55.5% | 0.1% |
| **BaMV + mCherry*i*** | 525,237,056 | 5.2% | 7.9% | 85.2% |
| **BaMV + imp α2*i*** | 512,721,366 | 2.3% | 4.1% | 92.1% |
