# Supplementary material for: Importin α2 participates in RNA interference against bamboo mosaic virus accumulation in Nicotiana benthamiana via NbAGO10a‐mediated small RNA clearance
Source: Mol Plant Pathol. 2024 Jan 19;25(1):e13422. doi: 10.1111/mpp.13422 (PMC10799208; doi:10.1111/mpp.13422)
Supplement: Supplementary file 9 — Table S3. List of primers in the experiments. [file MPP-25-e13422-s009.docx]

Table S3. List of primers in the experiments

| **Name** |  |  | **Sequence (5’→3’)** | **Note** |
| --- | --- | --- | --- | --- |
| **XbaI-Nbimp1-F** |  |  | CGTCTAGAAGAGAAGAAAGTTTGCTGAAGAAGCGT | VIGS |
| **XhoI-Nbimp1-R** |  |  | GGCTCGAGAAAGATATGATAGAGCCCAGCATGCATCTGT | VIGS |
| **XbaI-Nbimp2-F** |  |  | CGTCTAGAAGAGAAGAGAGTTTGCTCAAGAAGCGT | VIGS |
| **XhoI-Nbimp2-R** |  |  | GGCTCGAGAAAGGTAAGAAAGTGCCCAGCATGCATCTGT | VIGS |
| **EcoRI-impaCII F** |  |  | AAGAATTCTCTGGGCTTTGGGCAATGTTG | VIGS |
| **BamHI-impaCII R** |  |  | AAGGATCCCTGCCTCAATTACAGCCTGAATC | VIGS |
| **BamHI-impaCIII F** |  |  | AAGGATCCGCCTTCTCTCTAAGTCGGAATTC | VIGS |
| **XhoI-impaCIII R** |  |  | AACTCGAGGTAGGGCTCCTTGTAATAGGAG | VIGS |
| **XbaI-mCherry-F** |  |  | CGTCTAGATTCATGTACGGCTCCAAGGC | VIGS |
| **XhoI-mCherry-R** |  |  | GGCTCGAGCTGCTTGATCTCGCCCTTCA | VIGS |
| **Xba_TRV_HEN1_F** |  |  | AAAATCTAGAAGTTGAATTGGTCTAAGTA | VIGS |
| **XhoI_TRV_HEN1_R** |  |  | AAAACTCGAGTCCACAGTGAGATTATCTTCTTTA | VIGS |
| **EcoRI-BamHI-HA-modi-5'impa1-ABD-F** |  |  | AAAGAATTCGGATCCATGTACCCATACGATGTTCCAGATTACGCTATGTCGCTGAGGCCGAACTCGAGAAC | Y2H |
| **EcoRI-3'impa1-ABD-R3** |  |  | AAAGAATTCTCATGAACTGAAGTTGAATCCTCCTGATG | Y2H |
| **EcoRI-BamHI-5'impa2-ABD-F** |  |  | AAAGAATTCGGATCCATGTACCCATACGATGTTCCAGATTACGCTATGTCTCTGAGACCAAGTG | Y2H |
| **EcoRI-3'impa2-spe-ABD-R4** |  |  | GAATTCTCAACCAAACTTGAATCCACCAGAGGGAAG | Y2H |
| **EcoRI-BamHI-CP-F2** |  |  | AAGAATTCGGATCCATGTCTGGAGCTGGAACGGGA | Y2H, AD^a^ |
| **EcoRI-CP-R** |  |  | AAGAATTCTTAGTCTGATGTTGGTTCGGGAA | Y2H, AD^a^ |
| **EcoR1-CP-F** |  |  | AAGAATTCATGTCTGGAGCTGGAACGGGA | Y2H, BD^a^ |
| **SalI-CP-R2** |  |  | AAGTCGACTTAGTCTGATGTTGGTTCGGGAAGAG | Y2H, BD^a^ |
| **SalI-BamHI-TGBp1-F** |  |  | AAGTCGACGGATCCATGGATAACCGGATAACTGACC | Y2H, AD^a^ |
| **Srf1-TGBp1-F** |  |  | AAGCCCGGGCAATGGATAACCGGATAACTGACC | Y2H, BD^a^ |
| **SalI-TGBp1-R** |  |  | AAGTCGACTCAAGTGGTCTGGCCAGATGAA | Y2H |
| **BsiwINbAGO10aF** |  |  | AAAACGTACGAGATGCCTATAAGGCAGATGAAA | Expression |
| **XmaINbAGO10aR** |  |  | AAAACCCGGGCTAACAATAAAACATTACTCTCTTCAC | Expression |
| **imp1-RT-F** |  |  | CCATTGTGCGATCTTCTGGTTT | RT-qPCR |
| **imp1-RT-R** |  |  | TCAAGCCCTTCCAAGCAGACT | RT-qPCR |
| **imp2-RT-F** |  |  | TGATTTGCTTGTGTGCCCTGAT | RT-qPCR |
| **imp2-RT-R** |  |  | TCCTTCCAGACAGACGGTGACA | RT-qPCR |
| **QRT-impaC2F** |  |  | GCTGGCATGAATGGTGGAATTA | RT-qPCR |
| **QRT-impaC2R** |  |  | GTCCAATCCATCACATTCATCG | RT-qPCR |
| **QRT-impaC3F** |  |  | GCTGATGTTGAGGCAGCAAGAT | RT-qPCR |
| **QRT-impaC3R** |  |  | TTCGGCATTCCTCTCAAGACC | RT-qPCR |
| **NbHEN1F** |  |  | AGGCCTTATTCTCTCCCCCATT | RT-qPCR |
| **NbHEN1R** |  |  | CCACACCCAAAATCAACCAAAG | RT-qPCR |
| **NbAGO10a_F** |  |  | GCCTGCGCTAAAAGAAAAC | RT-qPCR |
| **NbAGO10a_R** |  |  | GAAAATAGAGCTGCTGTAC | RT-qPCR |
| **NbPR1_F** |  |  | TGGCTGCAGATTGTAACCTCG | RT-qPCR |
| **NbPR1_R** |  |  | GGTTTTCGCCGTATTGACCAT | RT-qPCR |
| **NbPR2_F** |  |  | GGAAATGAAGTTTCTCCCGGA | RT-qPCR |
| **NbPR2_R** |  |  | CAGGAGCAACAAATGGTGCA | RT-qPCR |

^a^AD and BD indicate the plasmids for yeast two-hybrid.
